# Mapping characteristics of mental skills training interventions in dance using TIDieR: a mixed-methods systematic review

**DOI:** 10.1136/bmjopen-2025-104552

**Published:** 2025-11-13

**Authors:** Michelle Schachtler Dwarika, Mary L Quinton, Sanna Nordin-Bates, Jennifer Cumming

**Affiliations:** 1 School of Sport, Exercise and Rehabilitation Sciences, University of Birmingham, Birmingham, UK; 2 Swedish School of Sport and Health Sciences GIH, Stockholm, Sweden

**Keywords:** MENTAL HEALTH, Systematic Review, SPORTS MEDICINE

## Abstract

**Abstract:**

**Objectives:**

Mental skills training (MST), which systematically uses techniques to build mental skills, is a popular intervention approach that may benefit dancers. However, information presented in existing MST interventions in dance is dispersed, making it difficult to offer evidence-based recommendations. To address this, the Template for Intervention Description and Replication (TIDieR) can improve transparency and replicability in intervention reporting, thus supporting researchers in assessing existing evidence and enhancing future intervention design. Guided by this framework, the aim of this mixed-methods systematic review was to provide an overview of existing MST interventions in dance and examine (1) the characteristics of effective MST interventions in dance and (2) how the reporting of these interventions aligned with the 12 TIDieR items.

**Design:**

Mixed-methods systematic review.

**Data sources:**

PsycInfo, Medline, Embase, SportDiscus, Web of Science and the first 30 pages of Google Scholar were searched from database inception until August 2024.

**Eligibility criteria:**

Quantitative, qualitative or mixed-methods approaches investigating MST interventions in which dancers used mental techniques, developed mental skills or enhanced mental qualities to improve physical and mental well-being.

**Data extraction and synthesis:**

Two reviewers independently screened identified studies in Covidence. Using the TIDieR framework, one reviewer extracted data while a second checked 30% of studies for accuracy. A convergent integrated synthesis was conducted.

**Results:**

Findings from the 21 included studies indicated that intervention effectiveness was determined by characteristics relating to both recipients and deliverers. While TIDieR items describing intervention content were most reported, few studies described fidelity.

**Conclusion:**

Future MST interventions in dance should consider multiple aspects of effectiveness and involve dancers and deliverers in mapping needs, values and outcomes. Structured reporting tools like TIDieR may enhance reporting clarity and intervention replicability.

**PROSPERO registration number:**

CRD42024537249.

**STRENGTHS AND LIMITATIONS OF THIS STUDY**

- This is the first mixed-methods systematic review of which we are aware that brings together quantitative and qualitative evidence on mental skills interventions in dance.
- To minimise publication bias and promote a balanced picture of evidence, the review included grey literature with clearly defined methods sections (eg, book chapters and theses/dissertations).
- By using the Template for Intervention Description and Replication (TIDieR) checklist as an extraction tool, this review offers researchers and practitioners suggestions on how to investigate existing evidence on interventions.
- Given the inconsistency in reporting of results across studies, further analysis (eg, meta-analysis) was not possible.
- The eligibility criteria and searches were skewed towards western concert dance styles.

## Introduction

 Mental skills training (MST; also called psychological skills training) is a popular intervention approach in sport and other performance domains and has been shown to be beneficial for improving individuals’ performance, self-development and mental well-being.^1 2^ The term MST entails the systematic use and exploration of techniques to build mental skills which reduce risk factors and enhance protective factors against the challenges performers face and navigate.^3 4^ MST literature in sport differentiates mental skills, mental qualities and mental techniques.^1^,^3^ Mental qualities can be seen as psychological characteristics (eg, self-awareness, robust confidence, psychological flexibility) that facilitate self-development, optimal performance and mental well-being.^2^,^4^ It is suggested that these mental qualities are achieved through the use of mental skills.^4 5^ Mental skills are active and deliberate actions (eg, maintaining confidence, managing difficult emotions) to self-regulate and manage one’s cognitions, emotions and behaviours.^4 5^ Mental techniques are tools or procedures (eg, imagery, goal-setting, grounding routines) that athletes use to regulate their mental state and develop mental skills.^4 6^ MST is therefore the process which facilitates the learning and implementation of mental techniques that assist the development of mental skills and mental qualities to achieve performance success and well-being.^3^

Originating in sport, MST interventions have been applied in different disciplines such as healthcare,^7 8^ the military^9^ and education,^9^ and show promise for also supporting performing artists, like dancers, in addressing challenges compromising their mental health and enhancing their well-being. For instance, Aujla and Farrer showed that freelance dance artists use mental skills (eg, maintaining confidence) to enhance mental qualities (eg, optimism, dedication and self-awareness), which supported their self-development, performance and well-being.^10^ Other literature suggests that mental skills could protect dancers’ mental well-being^11^ and that dance professionals perceive MST as beneficial to incorporate in dance practices.^12 13^ However, despite endeavours to develop MST interventions in dance, the existing evidence appears disparate and shows significant variations in research design and intervention characteristics (eg, content).^13^,^16^ For instance, research designs span from solely qualitative inquiries^13^ to mixed-method approaches^17 18^ and purely quantitative designs.^19^ Some interventions relied on an existing, sparse dance literature to shape their content,^16^ while others consulted with other interested parties (eg, dance teachers, school personnel) to inform their intervention.^13 20^ Consequently, researchers might struggle to determine which intervention characteristics are of importance when designing, developing or evaluating an MST intervention in dance.

Adopting the Template for Intervention Description and Replication (TIDieR) in this mixed-methods systematic review (MMSR) can offer dance researchers suggestions for understanding and making sense of this diverse evidence.^21^ By describing replicable and essential intervention characteristics (eg, who delivers the intervention, how and where the intervention is delivered, whether individual adaptations are used), the TIDieR offers support in assessing and using reported information.^21^,^24^ To exemplify, researchers designing a behaviour change programme to improve psychological well-being of the general population used the TIDieR for developing and reporting intervention elements, thus hoping to improve its replicability and relevance for public health literature.^25^ Cumming and Quinton^23^ used the TIDieR not only to inform the development of a theoretically grounded imagery intervention and improve its replicability, but also to protect participants from inadequately planned and ill-defined activities. Hence, the TIDieR is well suited to enhance dance researchers’ understanding of adequately reporting intervention characteristics and thereby improving the quality, replicability and sustainability of psychologically informed interventions in dance.^21 23 26^

### Objectives

The aim of this MMSR was to provide an overview of existing MST interventions in dance and examine their characteristics and effectiveness within the TIDieR framework. To achieve this objective, the following was investigated:

1. What are the characteristics of effective MST interventions in dance?
2. How does the reporting of these interventions align with the 12 TIDiER items and what, if any, reporting gaps are occurring?

## Methods

### Protocol and registration

The protocol of this MMSR was registered on PROSPERO (CRD42024537249) and has been published in 2024 in a peer-reviewed journal.^27^

The MMSR follows elements from the Joanna Briggs Institute’s (JBI) MMSR^28^ and the Preferred Reporting Items for Systematic reviews and Meta-Analyses (PRISMA) guidance.^29^ The researchers conducted the review from August 2024 to February 2025.

### Patient and public involvement

Patients or the public were not involved in this review.

### Eligibility criteria

Studies of interest included quantitative, qualitative or mixed-methods approaches and designs such as randomised controlled trials (RCTs), non-randomised interventions, quasi-experimental studies, case studies and any qualitative (eg, case studies, action research, ethnographies) and mixed-methods (eg, convergent, explanatory, sequential, exploratory sequential) research designs. To minimise publication bias and promote a balanced picture of evidence, grey literature with clearly defined method sections (eg, book chapters and theses/dissertations) was included in the review. All included studies were in English, French, German or Scandinavian languages.

The authors of this review used the population, intervention, comparison, outcome (PICO) principle to inform the eligibility criteria of this review.^28 30^

#### Population

Included studies involved dancers, dance students and dance professionals in any dance genre such as, but not limited to, ballet, contemporary, hip hop, modern and jazz dance. Participants included are (a) vocational dancers who dance pre-professionally at vocational dance schools, dance institutions, at college or university level; (b) professional dancers that either work on a freelance basis or are employed at a professional dance company and are performing regularly to a paying audience; (c) recreational dancers that engage in dance sessions in dance studios or other arenas that offer dance classes or (d) any type of dance leader (eg, dance educators, artistic directors) who works with any dancers, from recreational to professional level. The authors excluded individuals who engage in dance for health, rehabilitation or therapy approaches.

#### Intervention

Studies investigated MST interventions in which dancers used mental techniques (eg, imagery, goal setting) to acquire or improve mental skills (eg, emotion regulation) that, in turn, enhance mental qualities needed to improve physical and mental well-being. Interventions that were dance technique/motor skill focused were excluded.

#### Comparison

This aspect was not applicable to the current review as no control groups were compared.

#### Outcome

Relevant studies examined pre- to post-intervention changes in mental qualities, mental health and/or symptoms of common mental disorders (eg, depression, anxiety). ‘Mental health’ was defined according to Keyes’ (2002) dual continua model of mental health,^31^ which incorporates components of emotional well-being, psychological well-being and social well-being. Included studies measured pre- and post-intervention mental health and/or different dimensions of mental well-being with scales (eg, the Ryff psychological well-being scale^32^; the WHO well-being scale^33^) or evaluated well-being effects qualitatively (eg, observations, diaries).

Symptoms of common mental disorders in dancers, including (but not limited to) depression, anxiety and eating disorders, were either measured with scales (eg, the Beck Depression Inventory,^34^ the Sport Performance Anxiety Scale (SAS-2)^35^ or Eating Attitudes Test (EAT-26)^36^) or captured qualitatively (eg, observation, follow-up interviews) pre-and post-intervention.

Potential mechanisms by which the primary outcomes were achieved included changes in mental technique use/ability from pre- to post-intervention and whether these changes were associated with changes in mental qualities, mental health and/or mental illness symptomology.

### Information sources

The first author conducted electronic searches from inception of the database until August 2024 in PsycInfo (Ovid), Medline (Ovid), Embase (Ovid), SportDiscus (Ebsco) and Web of Science (Clarivate). A follow-up search was conducted by the first author in February 2025 and no new studies were found at that point.

### Search strategy

The search strategy was designed in consultation with a research librarian and adapted for each included information source. The search also included (a) the first 30 pages of the search engine Google Scholar and (b) citation chasing. All search strings are presented in table 1.

**Table 1:** Search strings

Database	Search string
PsycInfo	dance*.mp. OR ballet.mp. or exp Dance/ OR exp Dance/ or college dance.mp. OR exp Dance/ or vocational dance.mp AND exp Skill Learning/ or exp Performance/ or mental skills.mp. AND intervention.mp. or exp Intervention/ OR training.mp. or exp Training/ OR program.mp.
Medline	dance*.mp OR ballet.mp. or Dancing/ OR Dancing/ or Students/ or collegiate dance.mp. OR Students/ or Dancing/ or vocational dance.mp. AND Athletic Performance/ or mental skills.mp. OR Athletic Performance/ or psychological skills.mp. AND intervention.mp. OR training.mp. OR program.mp.
Embase	dance*.mp.OR dancing/ or vocational dance.mp OR ballet dancer/ or ballet.mp. or dancing/OR collegiate dancer.mp. AND mental skills.mp. or mental performance/ or skill/ OR skill/ or psychological skills.mp. AND Intervention.mp. or intervention study/ or psychosocial intervention/ OR program development/ or Program.mp. OR training/ or Training.mp.
Sportdiscus	AB dance* AND TI program OR TI training AND mental skills OR psychological skills AND AB intervention
Web of science	dance* (Topic) and Dance (Should – Search within topic) and Ballet (Should – Search within topic) and Dancing (Should – Search within topic) and Contemporary Dance (Should – Search within topic) and Dancers (Should – Search within topic) and Dance Education (Should – Search within topic) AND mental skills* (Topic) and Mental Skills (Should – Search within topic) and Coping Skills (Should – Search within topic) and Mental Training (Should – Search within topic) and Mental Skills Training (Should – Search within topic) and Psychological Skills (Should – Search within topic) OR psychological skills* (Topic) and Psychological Skills (Should – Search within topic) and Coping Skills (Should – Search within topic) and Mental Skills (Should – Search within topic) and Psychological Skills Training (Should – Search within topic) and Psychological Performance (Should – Search within topic) AND intervention (Topic) and Intervention (Should – Search within topic) and Interventions (Should – Search within topic) OR training (Topic) and Training (Search within topic) and Education (Should – Search within topic) OR program (Topic)
Google scholar	psychological interventions ballet mental skills intervention dance

### Data management

Covidence, a web-based systematic review platform developed to guide reviewers through the systematic review workflow, was used to screen, assess and extract the studies.^37^ Extraction and assessment tables and a PRISMA flow diagram were created and adapted in Covidence.^37^

### Selection process

**Figure 1:**
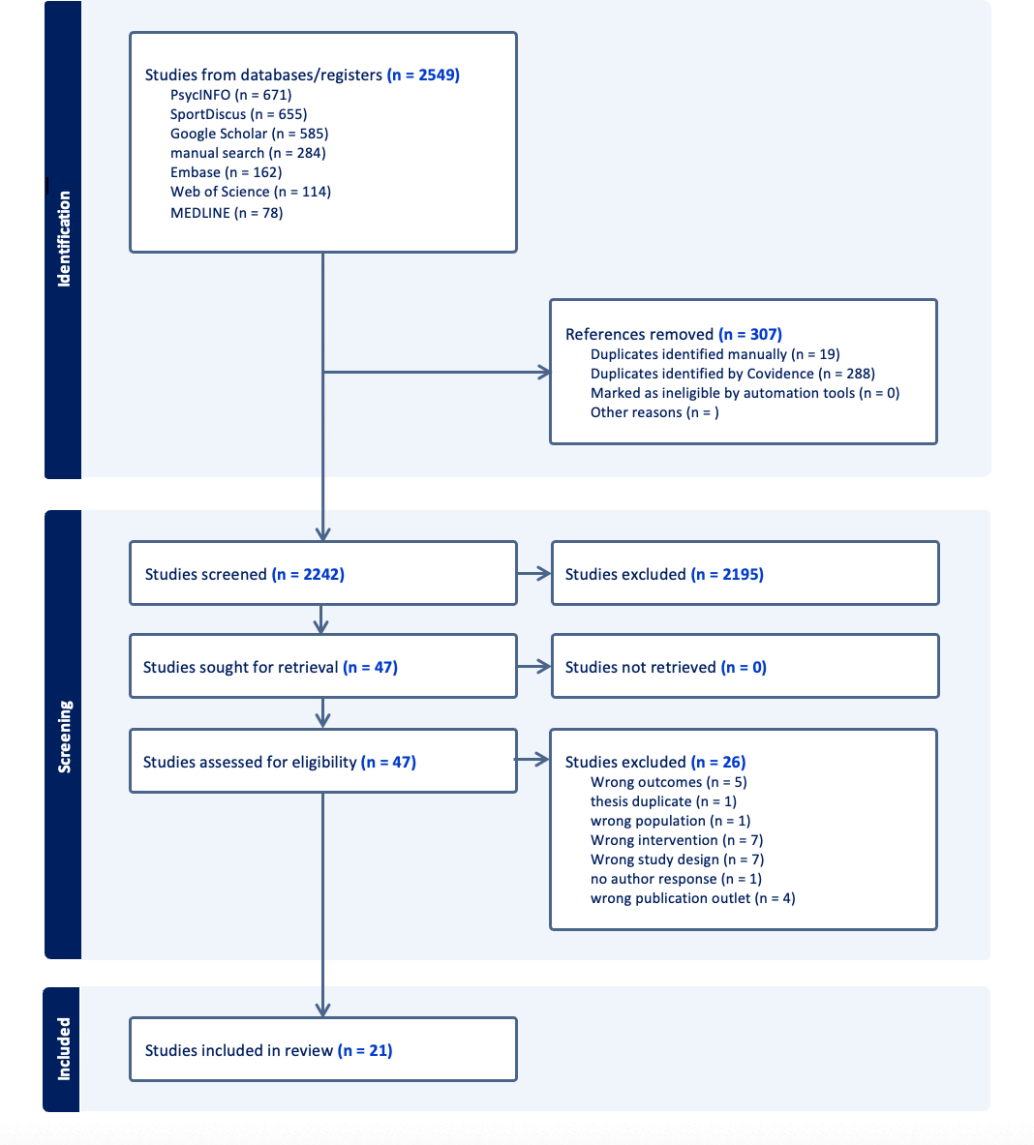
Preferred Reporting Items for Systematic reviews and Meta-Analyses (PRISMA) flow diagram.

Following the search, all identified studies were loaded into Covidence and duplicates removed. Titles and abstracts were screened by the first and fourth author for assessment against the inclusion criteria for the review. The full texts of selected citations were assessed in detail against the inclusion criteria and, based on the inclusion/exclusion criteria, sorted into ‘inclusion’, ‘exclusion’, ‘full text review’ or ‘irrelevant’. Reasons for exclusion of full text studies that did not meet the inclusion criteria were recorded and reported in the PRISMA flow diagram (see figure 1).

### Data extraction

The first author used the TIDieR framework and its 12 checklist headings (see online supplemental table S3) to extract data from studies included in the review.^21^ The use of the TIDieR headings allowed the authors to identify disparities and similarities of the studies’ documented intervention characteristics.^38^ Additionally, the first author extracted information about participants involved in each study (see online supplemental table S1) and the reported effectiveness of the interventions. The second author checked 30% of the extracted studies for accuracy. One author was contacted to request missing data. However, due to the data not having been formally published, the study was excluded from the review.

### Risk of bias assessment and confidence in cumulative evidence

The quality of all included studies, including those from the grey literature, was appraised using the Mixed Methods Appraisal Tool (MMAT).^39^ The first and second author independently rated each study’s possible risk of bias as “yes” “no” or, where insufficient detail was provided, as “can’t tell”. Disagreements that arose between the reviewers were resolved through discussion.

Levels of evidence (LoE) and the Confidence in the Evidence from Reviews of Qualitative Research (CerQual) were used to assess the strength of evidence of each study and integrated in the table assessing the studies’ methodological quality.^40 41^ To evaluate mixed-methods studies, the qualitative evidence was assessed with CerQual and the quantitative data with LoE. The results of critical appraisal and the certainty assessment can be accessed in online supplemental table S2.

### Data synthesis and integration

This review used a convergent integrated approach according to the JBI methodology for MMSR and followed three steps.^28^ In step one, the reviewers considered the final phases of both quantitative and qualitative evidence and examined whether findings naturally complemented each other. Then, quantitative data were qualitised (ie, transformed into textual descriptions) and combined with qualitative findings.^42^ In step two, the assembled data were pooled and structured according to the 12 TIDieR checklist items. In step three, the reviewers produced a preliminary narrative synthesis of the results. Findings were then structured and reported according to the 12 TIDieR checklist items. Data on intervention characteristics associated with improvements in dancers’ mental qualities, mental health and/or symptoms of common mental disorders (eg, depression, anxiety) were first synthesised and reported separately and then combined with the TIDieR evidence, the risk of bias and certainty assessment (see online supplemental table S5).

## Results

### Included studies and design

As presented in the PRISMA flow diagram (figure 1), the initial search identified 2549 studies. After the removal of 307 duplicates, 2242 titles and abstracts were screened in Covidence^37^ and 2195 studies were excluded in the process. The remaining 47 studies were reviewed in full text, which resulted in the exclusion of 26 investigations.

Of the final included studies (n=21), 12 (12/21) were peer-reviewed articles, 7 (7/21) were theses and 2 (2/21) were book chapters. The majority of the included studies were mixed-methods inquiries (9/21). Seven (7/21) were quantitative investigations of which 4 (4/7) were quantitative non-randomised, and 3 (3/7) were quantitative randomised. The remaining 5 (5/21) studies were qualitative investigations.

**Figure 2:**
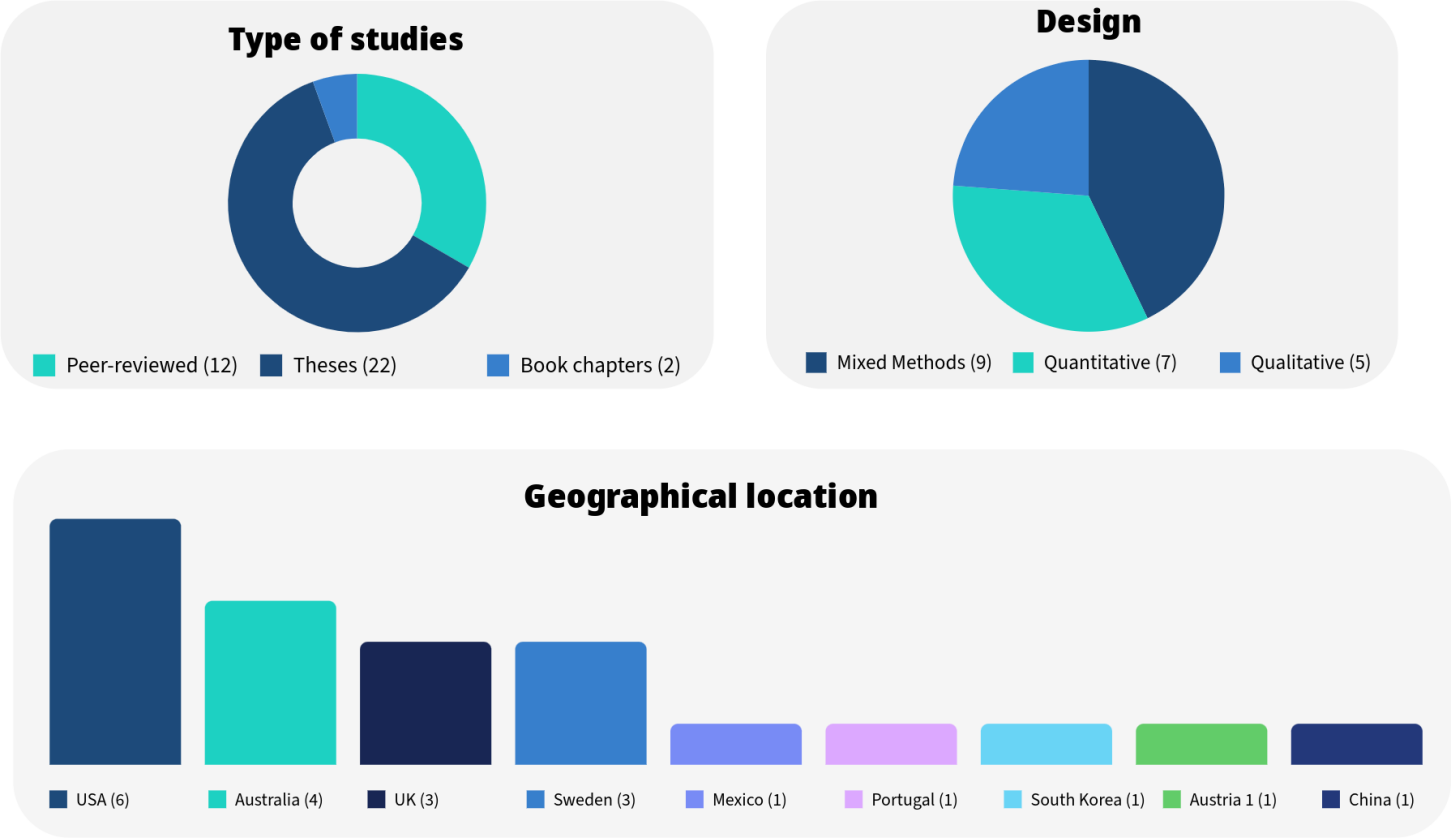
Overview of study characteristics.

Seven studies (7/21) were quasi-experimental designs, 3 (3/21) case studies, 3 (3/21) pilot studies, 3 (3/21) RCTs, 1 (1/21) observational, 1 (1/21) cohort and 1 (1/21) within-group design. Two investigations (2/21) did not specify their study design. As depicted in figure 2, 6 studies (6/21) were conducted in the USA, 4 (4/21) in Australia, 3 (3/21) in Sweden and the UK, and 1 (1/21) in Mexico, Portugal, South Korea, Austria and China respectively.

### Sample

A total of 855 dancers and 17 dance teachers are represented in this review. Of those, 607 (607/872) were female and 117 (117/872) were male. Of the total included participants, the gender of 148 (148/868) individuals was not specified, either because the article misreported participating dancers’ gender, participants did not disclose this information, or authors did not report the gender of their participants. Of the 855 dancers, 701 (701/855) were pre-professional (mean age=17.65, SD=3.60), 132 (132/855) were professional (mean age=22.13, SD=2.58) and 22 (22/855) were recreational (mean age=15.96, SD=1.6).

**Figure 3:**
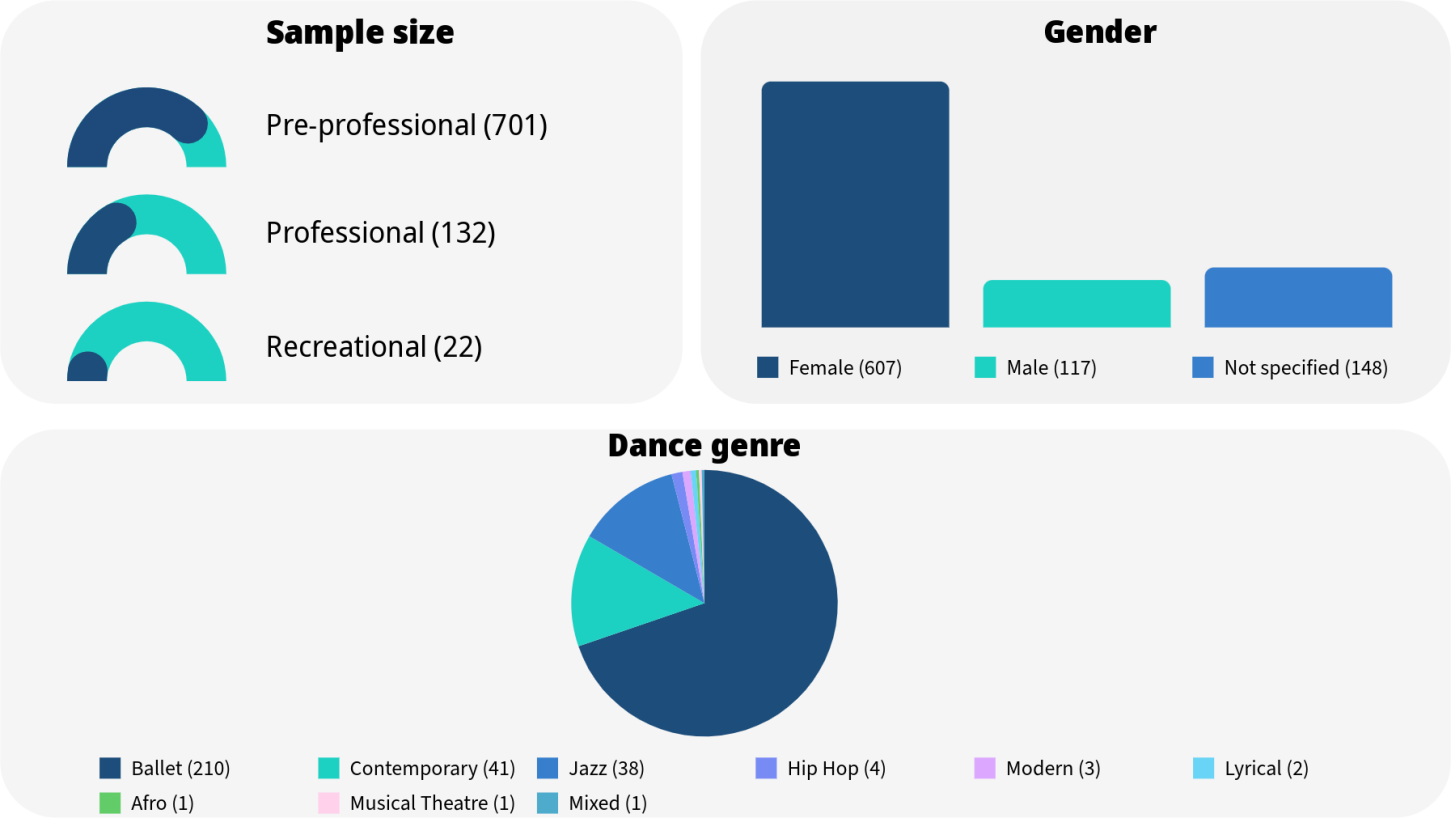
Overview of sample.

Only 13 studies (13/21) specified the dance genre of their participants, which included ballet (210/868), contemporary (41/868), jazz (38/868), hip hop (4/868), modern (3/868), lyrical (2/868), afro (1/868), musical theatre (1/868) and mixed (1/868). As eight studies (8/21) did not mention any dance genre, the dance style of the remaining 567 (567/868) participants is unknown. An overview of these findings can be seen in figure 3.

### Assessment of methodological quality

In line with MMAT guidelines,^39^ studies were appraised by “yes”, “no” and “can’t tell” responses. LoE^40^ and CerQual^43^ were used to assess the strength of evidence of each study. Mixed-methods studies (9/21) were assessed by evaluating the quantitative parts of the studies with LoE and the qualitative parts with CerQual. Generally, findings indicate moderately high to high risk of bias and moderate to low confidence in the studies included in this review (for a detailed overview, see online supplemental table S2).

### Effectiveness

Two of the included studies (2/21) did not specify whether the intervention was effective. Three studies (3/19) reported achieving intended outcomes in one or more dependent variables from pre- to post-intervention (significant p value of<0.05 in quantitative studies or equivalent description of such in qualitative studies). Nine studies (9/19) reported moderate effectiveness (ie, one or more dependent variables significantly changing from pre- to post-intervention in quantitative studies as reflected in a p value of<0.05, or in an equivalent description of such in qualitative studies). Seven (7/19) studies reported no change in the dependent variables. Of those three studies that reported achieving intended outcomes, two (2/3) were mixed-methods and one (1/3) was quantitative. Of the nine studies that reported moderate effectiveness, four (4/9) were quantitative, two (2/9) were mixed-methods and three (3/9) were qualitative investigations. Of the six studies that reported no effect, four (4/6) were mixed-methods and two (2/6) were quantitative studies.

Of the studies achieving intended outcomes, two (2/3) described changes in mental health outcomes (eg, anxiety, depression, well-being), and one (1/3) described changes in mental qualities (eg, self-awareness, self-esteem). Of the moderately effective studies, five (5/9) described changes in mental qualities, two (2/9) reported changes in mental health outcomes and two (2/9) measured changes in use of mental techniques (eg, imagery, self-talk) as the dependent variables.

Generally, findings suggest that studies achieving intended outcomes considered dancers’ individual needs, goals and motivation to participate,^44^ delivery style^13 44^ or acknowledged participants’ subjective experiences related to mental skills or qualities.^45 46^ For example, Lubert *et al*^44^ tailored their intervention sessions to performing artists’ needs by involving participants in determining desired outcomes and clarifying how the mental coach delivering the intervention could address their concerns. Once specific outcomes or needs were established, content and delivery were adjusted and tailored strategies incorporated which, in turn, contributed to decreasing the performing artists’ performance anxiety and increasing their mental well-being.^44^ As suggested in this example, intervention effectiveness is influenced not only by characteristics of recipients (eg, dancers’ needs, values and outcomes) but also by those of deliverers (eg, delivery style and interaction of situation and context).^47^ Thus, acknowledging dancers’ needs and perspectives, involving them in defining desired outcomes, and deliverers aligning their delivery styles to the individual’s needs and context appear crucial to enhance intervention effectiveness. For a more detailed overview of the included studies’ effectiveness, please see online supplemental table S3.

### Intervention reporting according to the TIDieR checklist

On average, researchers reported 9/12 TIDieR items, of which ‘When and How much’ (21/21), ‘Procedure’ (21/21), ‘Rationale’ (21/21) and ‘How’ (20/21) were the most recorded characteristics. Among the least reported were ‘Name’ (7/21), ‘Modifications’ (9/21) and ‘How well actually’ (10/21). This indicates that researchers described characteristics relating to intervention fidelity (eg, ‘How well’, ‘Modifications’) less frequently than those items relating to intervention content (eg, ‘Procedure’ and ‘Rationale’). For an overview of the TIDieR checklist results, see table 2, for a more detailed presentation of TIDieR reporting, see online supplemental table S4.

**Table 2:** Overview of reported TIDieR items

TIDieR item	Description	Reported items	Notes
Brief name	Name or title of the intervention	7/21	Missing or vague
Why	Rationale, theory or goal of the intervention	21/21	Strongly reported
What (materials)	Physical or informational materials used	15/21	Not clear how and where to access materials
What (procedures)	Procedures, activities and processes	21/21	Well reported
Who provided	Expertise, background and training of providers	18/21	Mostly researcher-led; few external collaborators
How	Modes of delivery (eg, face-to-face, online)	20/21	Mostly group-based, in-person
Where	Location of delivery	16/21	Often general or implied
When and how much	Number of sessions, schedule, duration	21/21	Clearly reported
Tailoring	Whether and how the intervention was personalised	14/21	Often insufficiently reported or only briefly mentioned
Modifications	Changes during the study	9/21	Rarely discussed
How well (planned)	Strategies to ensure fidelity	10/21	Underreported
How well (actual)	Extent to which intervention was delivered as planned	10/21	Fidelity assessments largely absent

The majority of researchers incorporated psychoeducational aspects in intervention content (9/21) and took a multimodal approach to MST (13/21), with imagery being the most used mental technique in sessions (13/21). To contrast, only a few studies targeted mental qualities (eg, self-esteem) and/or aspects of dancers’ mental health (eg, well-being, depression) rather than specific techniques (3/21) or underpinned their investigations with therapeutic or more overarching approaches (3/21) or themes (1/21). Interventions lasted between 5 days and 16 weeks and were mainly delivered by the lead investigators or researchers in group settings (13/18), with only a minority of the included studies (2/18) involving other interested parties (eg, teachers, health professionals) in the delivery of their sessions. In that regard, reporting on how these sessions and deliveries were tailored (14/21) or modified (12/21) often remained unclear or unspecified. Similarly, whether the interventions were carried out as intended and how adherence and delivery style was assessed were the most underreported TIDieR items in this review.

A joint overview of all reported items, intervention effectiveness and level of confidence in findings can be found in online supplemental table S5.

## Discussion

This MMSR presented an overview of characteristics of existing MST interventions in dance. To our knowledge, this is the first MMSR to (1) synthesise quantitative and qualitative evidence on MST interventions in dance and (2) use the 12-item TIDieR checklist to explore these characteristics in MST interventions targeting different dance populations. These novel contributions will not only advance knowledge on how MST interventions in dance have been conducted, but also offer suggestions on how the use of the TIDieR might be beneficial in planning and reporting more robust dance interventions.

### RQ1: characteristics of effective MST interventions in dance

Findings indicate that intervention effectiveness entails more than its intended outcome^48^ and might be influenced by a range of characteristics that not only relate to the person receiving the intervention (eg, needs, values and outcomes), but also to those delivering it (eg, delivery styles and perspectives, interaction between situation and context).^47^ Process evaluations providing insights into the mechanisms, processes and unexpected consequences of interventions can support researchers in mapping those characteristics and understanding why the intervention is (in)effective in its context.^47^,^49^ To exemplify, a process evaluation conducted by researchers aiming to increase physical activity in an after-school programme for children (the Bristol Girls Dance Project) showed that characteristics such as delivery style, deliverers’ needs and school context impacted the intervention’s effectiveness.^50^ However, findings in this review remain unclear as to whether researchers sufficiently acknowledged the importance of such characteristics to their programme’s effectiveness.^48^

Moreover, while studies included in the review generally considered characteristics relating to the dancers receiving the intervention (eg, participants’ demands, goals and subjective experiences of MST),^13 44 51^ none of the published manuscripts reported consulting with dancers and deliverers in their formative work to map characteristics essential to recipients and deliverers. Yet, existing research outside of dance shows that involving target users and deliverers in shaping the intervention according to their needs helped researchers to strengthen the meaningfulness, rigour and effectiveness of their intervention.^52^,^54^ For example, an MST intervention targeting youth at risk of homelessness (the MST4Life programme) consulted with their target users and deliverers from the get-go, which not only enabled the researchers to create intervention content tailored to users’ needs, but also to identify values and outcomes meaningful to those involved and targeted in the programme.^54 55^ Hence, participatory research or qualitative approaches involving dancers and programme deliverers in intervention development can enable researchers to take a more attuned approach to dancers’ and deliverers’ context, needs and values, and could be a promising avenue for future MST interventions.^56^

### RQ2: TIDieR reporting

While results demonstrated that researchers were adept at describing TIDieR items relating to intervention content (eg, ‘Procedure’ and ‘Rationale’), the findings also reveal a predominantly technique-oriented approach, where studies often reported the mental techniques included in the intervention (eg, imagery, self-talk, goal-setting), but often did not distinguish further between mental skills and qualities to be developed. Yet, the MST model by Holland *et al*^1^ shows that extending Vealey’s distinction between mental techniques and mental skills^2 6^ to include mental qualities into the MST framework might not only enhance our understanding of what individuals do (eg, performance), but also who they are (eg, their psychological well-being, identity and integrity).^1 4^ This broader conceptualisation implies that future MST interventions may require different theoretical underpinnings. For instance, a small number of studies in this review have explored the integration of established therapeutical models such as acceptance and commitment therapy and cognitive-behavioural therapy into MST interventions. These approaches may enhance dancers’ self-regulatory capacities and address the diverse challenges they encounter across performance, training and personal contexts.^57^ Although these avenues remain, to date, underexplored, they show promise in offering new approaches to content development attuned to dancers’ needs, values, goals and contexts, and can enhance the transparency of future intervention reporting.

Generally, studies reported characteristics relating to intervention fidelity (eg, “How well”, “Modifications”) less frequently. Fidelity concerns the implementation of an intervention’s key components and is assessed by describing aspects such as whether an intervention is delivered in line with a protocol, providing details of what has been delivered and how this was done, and outlining eventual changes made during intervention delivery or post-intervention.^57 58^ As fidelity assessments are vital to measure adherence to programme implementation and correctly attributing outcomes to interventions, descriptions of TIDieR items like “Modifications”, “Tailoring” and “How well” are essential to gain insight into an intervention’s fidelity and adherence.^21 58^ To exemplify, assessing barriers and enabling factors for a high-fidelity delivery style was done in a study evaluating an MST intervention with youth at risk for homelessness.^58^ Findings indicated that delivery styles (eg, need-thwarting vs need supporting) mattered to programme adherence and that characteristics related to fidelity (eg, ‘Modifications’, ‘How well’) were essential to adequately evaluate intervention outcomes.^58^

Tools like the TIDieR can facilitate more detailed and consistent fidelity reporting and could enable researchers to make adequate, evidence-based evaluations of an intervention’s outcomes and effectiveness.^54 58^ For example, the TIDieR would enable researchers to describe intervention characteristics in sufficient detail and raise awareness of characteristics (eg, items like ‘How well’ or ‘Modifications’) essential to intervention fidelity. Yet, findings from other systematic reviews using the TIDieR confirm that items describing fidelity (eg, ‘Modifications’, ‘How well actually’ and ‘Tailoring’) were not only less reported than other items but also described insufficiently to assess intervention efficiency.^2459^,^61^ Hence, while it can be argued that researchers might possess insufficient knowledge on fidelity assessments,^62^ or that aspects like ‘Modifications’ and ‘Tailoring’ are misunderstood as a lack of rigour,^58^ using the TIDieR to enhance intervention reporting will benefit researchers across disciplines and strengthen future MST interventions.

### Recommendations

The findings presented in this review have implications for researchers and practitioners developing MST interventions in dance. First, researchers conducting future MST interventions should not only undertake outcome but also process evaluations which will enable them to map fidelity, underpinning processes, mechanisms and characteristics that can explain intervention (in)effectiveness.^47^ Second, researchers should involve dancers and those delivering the programme more directly in the development of interventions. Acknowledging and implementing dancers’ and deliverers’ needs, values and requested outcomes will not only make the intervention more meaningful but also increase its effectiveness. Participatory research involving dancers^1^ in the formative work of intervention development will enable researchers to explore needs, values and outcomes and will, thus, be an important avenue to inform future MST interventions in dance. Third, a clearer conceptualisation of MST in dance that distinguishes between techniques, skills and qualities can support researchers in shaping meaningful and context-sensitive MST interventions in dance. Fourth, a standardised reporting tool like the TIDieR can help to prevent limitations identified in this review.^21 63^ Specifically, the TIDieR can support researchers in asking meaningful questions and accurately describing and implementing interventions, thus helping practitioners to address know-do gaps and get a clearer idea of how to translate research into practice.^47^ These suggestions may also be generalisable to other performance domains using MST, such as sport,^64^ healthcare^65^ and the military.^66^

### Strengths and limitations

This MMSR brings together quantitative and qualitative evidence on MST interventions in dance and makes several original contributions. First, the review presents an overview of MST interventions which can aid other researchers and practitioners to use lessons learnt to enhance the planning, conducting and reporting of their projects. Second, by using the TIDieR to disseminate and understand existing characteristics, it offers researchers suggestions on how to strengthen the designing and reporting of future MST interventions.

Yet, there are limitations to our findings. First, due to the focus of the review, several other interesting avenues, like MST interventions that were dance technique, motor skill or therapeutically focused, were excluded from this review. Choices like this inevitably impact the reviews’ comprehensiveness, but we believe that these areas deserve their own, dedicated review to serve different interests and target audiences in the dance research community. Second, studies had a moderate to high risk of bias which influences the strengths of our findings. Finally, despite the diversity of dance genres and their popularity around the world, non-western concert styles were under-represented in this review and deserve more dedicated attention in future research.

## Conclusion

This systematic review aimed to present an overview of existing quantitative and qualitative evidence on MST interventions in dance and used the 12 TIDieR items to explore characteristics essential to MST interventions’ effectiveness. Findings implied that future MST interventions can be further strengthened and tailored by using MST with greater conceptual clarity, considering characteristics relating to the person receiving and those delivering the intervention and involving dancers more directly in mapping and identifying needs, values and outcomes of MST interventions. By applying the TIDieR checklist as an extraction tool, this review provides an overview of existing evidence on interventions and identifies knowledge and reporting gaps related to fidelity. While these results imply that researchers should enhance the reporting and description of fidelity, they also indicate that standardised tools like the TIDieR can aid researchers in addressing knowledge gaps and, thus, facilitate more consistent and adequate reporting of future MST interventions.

## Supplementary material

10.1136/bmjopen-2025-104552 online supplemental table 1

10.1136/bmjopen-2025-104552 online supplemental table 2

10.1136/bmjopen-2025-104552 online supplemental table 3

10.1136/bmjopen-2025-104552 online supplemental table 4

10.1136/bmjopen-2025-104552 online supplemental table 5

## Data Availability

Data are available upon reasonable request.

## References

[1] Holland MJG, Woodcock C, Cumming J (2010). Mental Qualities and Employed Mental Techniques of Young Elite Team Sport Athletes. J Clin Sport Psychol.

[2] Vealey RS (2024). A framework for mental training in sport: Enhancing mental skills, wellbeing, and performance. J Appl Sport Psychol.

[3] Vealey RS (2007). Mental skills training in sport.

[4] Holland MJ, Cooley SJ, Cumming J (2017). Sport psychology for young athletes.

[5] Sharp L-A, Woodcock C, Holland MJG (2013). A Qualitative Evaluation of the Effectiveness of a Mental Skills Training Program for Youth Athletes. Sport Psychol.

[6] Vealey RS (1988). Future Directions in Psychological Skills Training. Sport Psychol.

[7] Anton NE, Bean EA, Hammonds SC (2017). Application of Mental Skills Training in Surgery: A Review of Its Effectiveness and Proposed Next Steps. Journal of Laparoendoscopic & Advanced Surgical Techniques.

[8] Bartels SJ, Forester B, Mueser KT (2004). Enhanced skills training and health care management for older persons with severe mental illness. Community Ment Health J.

[9] McCrory P, Cobley S, Marchant P (2013). The Effect of Psychological Skills Training (PST) on Self-Regulation Behavior, Self-Efficacy, and Psychological Skill Use in Military Pilot-Trainees. Mil Psychol.

[10] Aujla I, Farrer R (2015). The role of psychological factors in the career of the independent dancer. Front Psychol.

[11] Estanol E, Shepherd C, MacDonald T (2013). Mental Skills as Protective Attributes Against Eating Disorder Risk in Dancers. J Appl Sport Psychol.

[12] Klockare E, Gustafsson H, Nordin-Bates SM (2011). An interpretative phenomenological analysis of how professional dance teachers implement psychological skills training in practice. Research in Dance Education.

[13] Carattini CM (2020). Psychological Skills in Ballet Training: An Approach to Pedagogy for the Fulfilment of Student Potential.

[14] Skvarla LA, Clement D (2019). The Delivery of a Short-Term Psychological Skills Training Program to College Dance Students: A Pilot Study Examining Coping Skills and Injuries. J Dance Med Sci.

[15] Noh Y-E, Morris T, Andersen MB (2007). Psychological intervention programs for reduction of injury in ballet dancers. Res Sports Med.

[16] Klockare E (2014). A psychological skills training program for dancers: evaluation of the dancers’ use of psychological skills training techniques and possible effects of the program.

[17] Gerena C (2015). Positive Thinking in Dance: The Benefits of Positive Self-Talk Practice in Conjunction with Somatic Exercises for Collegiate Dancers.

[18] Peris-Delcampo D, Taipe-Nasimba N, Expósito V (2019). Psychological intervention using motivational coaching in dance sport: a single case study. BEHAVIORAL SCIENCES.

[19] Jeong EH (2012). The Application of Imagery to Enhance’flow State’in Dancers.

[20] Nordin-Bates SM, Lundström P, Melin AK (2023). Preventing Disordered Eating in Teenage Ballet Students: Evaluation of DancExcellent, a Combined CBT and Nutrition Education Intervention. Med Probl Perform Art.

[21] Hoffmann TC, Glasziou PP, Boutron I (2014). Better reporting of interventions: template for intervention description and replication (TIDieR) checklist and guide. BMJ.

[22] Madden SK, Cordon EL, Bailey C (2020). The effect of workplace lifestyle programmes on diet, physical activity, and weight-related outcomes for working women: A systematic review using the TIDieR checklist. Obes Rev.

[23] Cumming J, Quinton ML (2022). Improving the reporting of sport imagery interventions with TIDieR. Asian Journal of Sport and Exercise Psychology.

[24] Dijkers MP (2021). Overview of Reviews Using the Template for Intervention Description and Replication (TIDieR) as a Measure of Trial Intervention Reporting Quality. Arch Phys Med Rehabil.

[25] Jones M, Metse AP, Watkins A (2024). “EMERALD” online early intervention programme for psychological well-being: A detailed description using the TIDieR checklist. Digit Health.

[26] Ely FO, O. J, Munroe-Chandler KJ (2021). How Intervention Research Designs May Broaden the Research-to-Practice Gap in Sport Psychology. J Sport Psychol Action.

[27] Dwarika MS, Quinton ML, Nordin-Bates S (2024). Characteristics of mental skills interventions in dance: a mixed methods systematic review protocol. BMJ Open.

[28] Lizarondo L, Stern C, Carrier J (2019). Mixed methods systematic reviews.

[29] Brennan SE, Munn Z (2021). PRISMA 2020: a reporting guideline for the next generation of systematic reviews. JBI Evid Synth.

[30] Shamseer L, Moher D, Clarke M (2015). Preferred reporting items for systematic review and meta-analysis protocols (PRISMA-P) 2015: elaboration and explanation. BMJ.

[31] Keyes CLM (2002). The mental health continuum: from languishing to flourishing in life. J Health Soc Behav.

[32] Ryff CD (1995). Psychological Well-Being in Adult Life. Curr Dir Psychol Sci.

[33] World Health Organization (2024). WHO-five well-being index—background information. https://www.who.int/publications/m/item/WHO-UCN-MSD-MHE-2024.01.

[34] Jackson-Koku G (2016). Beck Depression Inventory. OCCMED.

[35] Smith RE, Smoll FL, Cumming SP (2006). Measurement of Multidimensional Sport Performance Anxiety in Children and Adults: The Sport Anxiety Scale-2. J Sport Exerc Psychol.

[36] Garner DM, Olmsted MP, Bohr Y (1982). The eating attitudes test: psychometric features and clinical correlates. Psychol Med.

[37] Kellermeyer L, Harnke B, Knight S (2018). Covidence and Rayyan. Jmla.

[38] Doyle F, Freedland K, Carney R (2019). Network meta-analysis of randomised trials of pharmacological, psychotherapeutic, exercise and collaborative care interventions for depressive symptoms in patients with coronary artery disease: hybrid systematic review of systematic reviews protocol. Syst Rev.

[39] Hong QN, Pluye P, Fàbregues S (2018). Mixed methods appraisal tool (MMAT), version 2018. Registration of Copyright.

[40] Global S (2015). Definition of levels of evidence (LoE) and overall strength of evidence (SoE). Global Spine J.

[41] Munn Z, Tufanaru C, Aromataris E (2014). JBI’s systematic reviews: data extraction and synthesis. Am J Nurs.

[42] Tibbs M, O’Reilly A, Dwan O’Reilly M (2022). Online synchronous chat counselling for young people aged 12-25: a mixed methods systematic review protocol. BMJ Open.

[43] Lewin S, Booth A, Glenton C (2018). Applying GRADE-CERQual to Qualitative Evidence Synthesis Findings: Introduction to the Series.

[44] Lubert VJ, Nordin-Bates SM, Gröpel P (2023). Effects of tailored interventions for anxiety management in choking-susceptible performing artists: a mixed-methods collective case study. Front Psychol.

[45] Stackpole AI, Quiroga-Garza A (2023). Overcoming Stage Anxiety with a Solution-Focused Approach. Journal of Dance Education.

[46] Kaplan JF (2014). Gaining Control of the Dancer in the Mirror: A Prevention Program for Recreational Ballet Students.

[47] Graham Moore SA, Barker M, Bond L (2015). Complex interventions in health: an overview of research methods.

[48] Skivington K, Matthews L, Simpson SA (2021). A new framework for developing and evaluating complex interventions: update of Medical Research Council guidance. BMJ.

[49] Tidmarsh G, Thompson JL, Quinton ML (2022). Process Evaluations of Positive Youth Development Programmes for Disadvantaged Young People: A Systematic Review. JYD.

[50] Sebire SJ, Edwards MJ, Kesten JM (2016). Process evaluation of the Bristol girls dance project. BMC Public Health.

[51] Moyle GM (2016). Mindfulness and dancers.

[52] Morrison L, Muller I, Yardley L (2018). The person-based approach to planning, optimising, evaluating and implementing behavioural health interventions. The European Health Psychologist.

[53] Muller I, Santer M, Morrison L (2019). Combining qualitative research with PPI: reflections on using the person-based approach for developing behavioural interventions. Res Involv Engagem.

[54] Cumming J, Whiting R, Parry BJ (2022). The My Strengths Training for Life™ program: Rationale, logic model, and description of a strengths-based intervention for young people experiencing homelessness. Eval Program Plann.

[55] Quinton ML, Tidmarsh G, Parry BJ (2022). A Kirkpatrick Model Process Evaluation of Reactions and Learning from My Strengths Training for Life. Int J Environ Res Public Health.

[56] Petts L, McGill A (2024). Disrupting the Obligation of Objective Knowledge in Dance Science Research. J Dance Med Sci.

[57] An M, Dusing SC, Harbourne RT (2020). What Really Works in Intervention? Using Fidelity Measures to Support Optimal Outcomes. Phys Ther.

[58] Tidmarsh G, Whiting R, Thompson JL (2022). Assessing the fidelity of delivery style of a mental skills training programme for young people experiencing homelessness. Eval Program Plann.

[59] Hudek N, Carroll K, Semchishen S (2024). Describing the content of trial recruitment interventions using the TIDieR reporting checklist: a systematic methodology review. BMC Med Res Methodol.

[60] Lim S, Liang X, Hill B (2019). A systematic review and meta‐analysis of intervention characteristics in postpartum weight management using the TIDieR framework: A summary of evidence to inform implementation. Obes Rev.

[61] James J, Costello JT, Drahota AK (2024). A benchmark for the quality of reflexology intervention reporting using the template for intervention description and replication (TIDieR) checklist: A systematic review. Eur J Integr Med.

[62] Toomey E, Hardeman W, Hankonen N (2020). Focusing on fidelity: narrative review and recommendations for improving intervention fidelity within trials of health behaviour change interventions. Health Psychol Behav Med.

[63] Yardley L, Ainsworth B, Arden-Close E (2015). The person-based approach to enhancing the acceptability and feasibility of interventions. Pilot Feasibility Stud.

[64] Barker JB, Slater MJ, Pugh G (2020). The effectiveness of psychological skills training and behavioral interventions in sport using single-case designs: A meta regression analysis of the peer-reviewed studies. Psychol Sport Exerc.

[65] van Agteren J, Iasiello M, Lo L (2018). Improving the wellbeing and resilience of health services staff via psychological skills training. BMC Res Notes.

[66] Adler AB, Bliese PD, Pickering MA (2015). Mental skills training with basic combat training soldiers: A group-randomized trial. J Appl Psychol.

