# Supplementary material for: Mapping characteristics of mental skills training interventions in dance using TIDieR: a mixed-methods systematic review
Source: BMJ Open. 2025 Nov 13;15(11):e104552. doi: 10.1136/bmjopen-2025-104552 (PMC12625948; doi:10.1136/bmjopen-2025-104552)
Supplement: online supplemental table 1 [file bmjopen-15-11-s001.pdf]

Table S1

Reference	Study title	Country	Population	Design	Methods	Journal
Andersen (2009)	The "canon" of psychological skills training for enhancing performance	USA	1 pre-professional, male ballet dancer, age not specified.	Case study	Qualitative	Book chapter
Carattini (2020)	Psychological skills in ballet training: An approach to pedagogy for<br>the fulfilment of student potential	Australia	17 female ballet teachers.	Observational (analysis of reflectiv	Qualitative	Thesis
Castillo (2018)	Implementation and evaluation of a performance profile intervention with collegiate dancers: A randomized controlled trial	USA	44 female collegiate dancers (Mean age = 19.82, SD = 3.00).<br><br>Twenty-eight (63.6%) participants were Caucasian, six (13.6%) were Hispanic, five (11.4%) were<br>multiethnic/mixed, four (9.1%) were African American, and one<br>(2.3%) identified as "other."<br><br>Dance style varied and included jazz (n = 9), ballet (n = 10),<br>contemporary (n = 14), modern (n = 3), hip-hop (n = 4), lyrical (n = 2), acro/tumbling (n = 1),<br>and musical theater (n = 1).	Randomized controlled trial	Quantitative	Electronic Theses, Treatises and Dissertations (thesis)
Fryer (2018)	The cascading effect: Mitigating the effects of choking under pressure in dancers	USA	23 pre-professional female dancers (Mean age= 11.63, SD= 2.66; Mexperience= 7.52, SD= 3.26) from a<br>local dance studio in Texas.	Randomized pretest-posttest<br>control group experiment and<br>semi-structured interviews	Mixed methods	Thesis
Gerena (2015)	Positive thinking in dance: The benefits of positive self-talk practice in conjunction with somatic exercises for collegiate d	USA	6 collegiate dancers (4 women, 2 men) Mean age= 18,6.	Pilot study	Mixed methods	Thesis
Harrison et al. (2024)	A 6-Week Mindfulness-Acceptance-Commitment Intervention to Improve Professional Ballet Dancers' Psychological<br>States Around Performance	Australia	16 professional ballet dancers (2 men and 14 women), aged between 19 and 35 years (Mean age = 25 years, SD = 4.88) .	Randomized controlled trial	Mixed methods	Journal of Dance Medicine & Science
Jeong (2012)	The application of imagery to enhance'flow state'in dancers	Australia	64 professional dancers, of both genders, but predominantly female<br>(female = 57; male = 7), who ranged in age from 17 to 28 years (Mean age = 20; SD = 2.46).	Quasi-experimental	Quantitative	Thesis
Kaplan (2014)	Gaining control of the dancer in the mirror: A prevention program for recreational ballet students	USA	22 female recreational ballet dancers (in their studios)<br><br>11 recreational dancers in the intervention group and 11 recreational dancers in the<br>control group. All participants were female. The participants ranged in age from 14-18<br>years old (Mean age = 15.96, SD = 1.6). Fourteen participants (63.6%) identified as White, three<br>participants (13.6%) as Multiracial, two participants (9.1%) as Asian, two participants<br>(9.1%) as Hispanic, and one participant (4.5%) identified as "Other".	Pilot study	Quantitative	Thesis
Karin & Nordin-Bates (2020)	Enhancing creativity and managing perfectionism in dancers through<br>implicit learning and sensori-kinetic imagery	Sweden	13 pre-professional ballet dancers (eight female, five male) at an elite ballet school.<br>They were Mean age= 14.46 years old (SD = .52).	Quasi-experimental	Mixed methods	Journal of Dance Education
Klockare (2014)	A Psychological Skills Training Program for Dancers:<br>Evaluation of the Dancers' Use of Psychological Skills Training Techniques and Possible Effects of the Program	Sweden	51 professional dancers (45 female and six male) ranging in age from 18 to 26 years (Mean age = 21.4 years, SD = 2.1).<br>The most common main dance style jazz (60%), followed by contemporary dance (33%) and ballet (7%).	Not specified	Quantitative	Thesis
Lubert et al. (2023)	Effects of tailored interventions for anxiety management in choking-susceptible<br>performing artists: a mixed-methods collective case study	Austria	2 female contemporary pre-professional dancers age (Mean age=23).	Collective case study	Mixed methods	Frontiers in Psychology
May et al. (2020)	Enhancing creativity by training metacognitive skills in mental imagery	UK	240 pre-professional dancers, 111 to the control group in the 2015-16 academic session (76 females,<br>68 %; 24 males, 22 %; 11 did not state sex), and 129 to the imagery group (103 females, 80 %, 24 males,<br>19 %; 2 did not state sex).<br><br>Ages at recruitment overall ranged from 17.9 years to 28.2 years, with a median of 19.0,<br>and did not differ between groups t(238) = 0.25, p = .800 or institution t(238) = 0.44, p = .662.	Cohort-based design	Quantitative	Thinking Skills and Creativity
Moyle (2016)	Mindfulness and dancers	Australia	96 pre-professional dance students undertaking full-time dance<br>training in either the BFA (Dance) or BFA (Dance Performance) course.	Pilot design	Mixed methods	Cambridge books online
Noh et al. (2007)	Psychological intervention programs for reduction of injury in ballet dancers	South Korea	45 female pre-professional ballet dancers aged 14 to 19 years (Mean age= 16.77, SD=1.37)	Quasi experimental.	Quantitative	Research in Sports Medicine
Nordin-Bates et al. (2023)	Preventing disordered eating in teenage ballet students: evaluation of dancexcellent,<br>a combined CBT and nutrition education intervention	Sweden	40 pre-professional ballet dancers Year 9 became a pilot group (n = 15) and years 7–8<br>the intervention group (n = 25).<br><br>Participants were 12-15 years old (Mean age = 13.90, SD = .84) and mostly female (77.5%)- 31 male=9.	Within-group design (with participa	Mixed methods	Medical problems of performing artists
Petsilas & Massarelli (2024)	Evaluating the impact and effectiveness of meditation in a dance conservatoire context	UK	15 second-year undergraduate dance students (no age or gender specified).	Not specified	Qualitative	Journal of Dance & Somatic Practices
Ritchie & Brooker (2018)	Imaging the future: an autoethnographic journey of using a guided and<br>cognitive-specific imagery intervention in undergraduate release-based contemporary dance technique	UK	8 Undergraduate ballet students (pre-professional), no age or gender specified.	Quasi-experimental drawing on pre	Qualitative	Research in Dance Education
Santos et al. (2022)	"Performing emotions and suffering": Recovery of depressive symptoms involving adolescent intervention in art and danc	Portugal	122 adolescents. 89 female, 33 male ; age (Mean age = 14.07; SD = 1.54).	Quasi-experimental design	Quantitative	European Journal of Mental Health
Skvarla & Clement (2019)	The Delivery of a Short-Term Psychological Skills Training Program to<br>College Dance Students: A Pilot Study Examining Coping Skills and Injuries	USA	30 collegiate dance students; male (N = 4) and female (N = 26) Their ages ranged from 18 to 22 years<br>(Mean age= 19.77, SD = 1.45).<br><br>77% of dancers (N = 23) described it as ballet-based and 23% (N = 7) as mixed-repertoire.	Quasi-experimental design	Mixed methods	Journal of Dance Medicine and Science
Stackpole & Quiroga-Garza (202	Overcoming Stage Anxiety with a Solution-Focused Approach	Mexico	16 pre-professional dancers classical (n = 8) and contemporary (n = 8) from a dance institute in the city of Monterrey, Mexico. 12<br>participants were female and six were male. The mean age of the participants was 17.81 years (SD = 3.14).<br><br>Number of participants or gender misreported!!	Quasi-experimental	Mixed methods	Journal of Dance Education
Zhang (2022)	Psychological Interventions to Performance Enhancement in Dance: A Hypothetical Case Study	China	23 year old female, first year in a provincial level professional dance company of Chin with high ambition to dance<br>professionally upon graduation.	Hypothetical case study	Qualitative	The Frontiers of Society

Summary	Geographical location	Population	Research design	Methods	Publication
	N= 21<br><br>6 (6/21) conducted in the USA (Andersen, 2009; Castillo, 2018; Fryer, 2018; Gerena, 2015; Kaplan, 2014; Skvarla & Clement, 2019)<br><br>4 (4/21) conducted in Australia (Carattini, 2020; Harrison et al., 2024; Jeong, 2012; Moyle, 2016)<br><br>3 (3/21) coducted in Sweden (Karin & Nordin-Bates, 2020; Klockare, 2014; Nordin-Bates et al., 2023)<br><br>1(1/21) conducted in Austria (Lubert et al., 2023), China (Zhang, 2022), Portugal (Santos et al., 2022), Mexico (Stackpole & Quiroga-Garza, 2023), and South Korea (Noh et al., 2007) respectively	N= 21<br><br>17 (17/21) studies reported the gender of their population (Andersen, 2009; Carattini, 2020; Castillo, 2018; Fryer, 2018; Gerena, 2015; Harrison et al., 2024; Jeong, 2012; Kaplan, 2014; Karin & Nordin-Bates, 2020; Klockare, 2014; Lubert et al., 2023; May et al., 2020; Noh et al., 2007; Nordin-Bates et al., 2023; Santos et al., 2022; Skvarla & Clement, 2019; Stackpole & Quiroga-Garza, 2023; Zhang, 2022)<br><br>3 (3/21) studies did not report the gender of their population (Moyle, 2016; Petsilas & Massarelli, 2024; Ritchie & Brooker, 2018)<br>1 (1/21) misreported the gender of their population (Stackpole & Quiroga-Garza, 2023)<br><br>*Level of participation*<br>Yes (N=19)<br>No (N=2)<br><br>Pre-professional (N= 14)<br>Professional (N= 3)<br>Recreational (N=1)	7(7/21) were quasi-experimental designs (Jeong, 2012; Karin & Nordin-Bates, 2020; Noh et al., 2007; Ritchie & Brooker, 2018; Santos et al., 2022; Skvarla & Clement, 2019; Stackpole & Quiroga-Garza, 2023)<br><br>3 (3/21) case studies (Andersen, 2009; Lubert et al., 2023; Zhang, 2022<br><br>3 (3/21) pilot studies (Gerena, 2015; Kaplan, 2014; Moyle, 2016)<br><br>3 (3/21) randomized-controlled trials (Castillo, 2018; Fryer, 2018; Harrison et al., 2024)<br><br>1 (1/21) observational (Carattini, 2020)<br><br>1 (1/21) cohort (May et al., 2020)<br><br>1 (1/21) within-group design (Nordin-Bates et al., 2023)<br><br>2 (2/21) did not specify their study design (Klockare, 2014; Petsilas & Massarelli, 2024).	The majority of the included studies were mixed methods inquiries (9/21) (Fryer, 2018; Gerena, 2015; Harrison et al., 2024; Karin & Nordin-Bates, 2020; Lubert et al., 2023; Moyle, 2016; Nordin-Bates et al., 2023; Skvarla & Clement, 2019; Stackpole & Quiroga-Garza, 2023).<br><br>7 (7/21) were quantitative investigations of which 4 (4/7) were quantitative non randomized (Klockare, 2014; May et al., 2020; Noh et al., 2007; Santos et al., 2022), and 3 (3/7) were quantitative randomized (Castillo, 2018; Jeong, 2012; Kaplan, 2014).<br><br>The remaining 5 (5/21) studies were qualitative (Andersen, 2009; Carattini, 2020; Petsilas & Massarelli, 2024; Ritchie & Brooker, 2018; Zhang, 2022) investigations.	12 (12/21)were peer-reviewed articles (Harrison et al., 2024; Karin & Nordin-Bates, 2020; Lubert et al., 2023; May et al., 2020; Noh et al., 2007; Petsilas & Massarelli, 2024; Ritchie & Brooker, 2018; Santos et al., 2022; Skvarla & Clement, 2019; Stackpole & Quiroga-Garza, 2023; Zhang, 2022),<br><br>7 (7/21) were theses (Carattini, 2020; Castillo, 2018; Fryer, 2018; Gerena, 2015; Jeong, 2012; Kaplan, 2014; Klockare, 2014)<br><br>2(2/21) were book chapters (Andersen, 2009; Moyle, 2016).
