# Supplementary material for: Mapping characteristics of mental skills training interventions in dance using TIDieR: a mixed-methods systematic review
Source: BMJ Open. 2025 Nov 13;15(11):e104552. doi: 10.1136/bmjopen-2025-104552 (PMC12625948; doi:10.1136/bmjopen-2025-104552)
Supplement: online supplemental table 3 [file bmjopen-15-11-s003.pdf]

Table S3

Reference	Change achieved	Quantitative findings: significant and desired direction of change in DV from pre- to post-intervention	Qualitative findings representing indicated effectiveness
Andersen (2009)	Not specified	Not specified	Not specified
Carattini (2020)	Moderate change	No quantitative evidence available	Targeted outcome: improving the use of PST in ballet class<br><br>"For some teachers, the trial significantly increased their implementation of PST and increased their students' performance and well-being, for others it didn't."
Castillo (2018)	Moderate change	*Dependent variables targeted: self-awareness and behavior*<br><br>Self-awareness:<br>Significant changes were seen for self-awareness (p < .05)<br>Behavior:<br>No statistically significant changes were seen for behavior (p = .06.)	No qualitative evidence available
Fryer (2018)	No change	*Dependent variables targeted: performance, self-efficacy, cognitive anxiety, somatic anxiety, self-confidence, self-consciousness*<br><br>Self efficacy<br>No statistically significant changes were seen for self-efficacy (p = 0.1)<br>Cognitive anxiety<br>No statistically significant changes were seen for cognitive anxiety (p = 0.95)<br>Somatic anxiety<br>No statistically significant changes were seen for somatic anxiety (p = 0.31)<br>Self-confidence,<br>No statistically significant changes were seen for self-confidence (p = 0.72)<br>Self-consciousness,<br>No statistically significant changes were seen for self-consciousness (p = 0.42)	"Overall dancers continued to rate their performance as moderate to excellent."
Gerena (2015)	Moderate change	*Dependent variables targeted: self-talk (improve focus, motivation, and being able to adapt current skills to new situations), thought occurence*<br><br>Self talk:<br>Most notably, the practice of self-talk improved for all subjects as well as an overall increased use of positive self-talk<br>Thought occurence:<br>A decrease in thoughts occurred post-study for all for participants signifying a potential improvement in focus and use of PST. On average, there was a 36% change in the number of total thoughts selected by participants (Individual percent change: A = 27%; B = 77%, C = 33%; D = 14%; E = 9%; F = 57%).	"It was discovered that all four aspects of psychological skills training (goal-setting, imagery, self-talk and relaxation) benefitted students' management of these 45 environmental stressors. The development and practice of self-talk cues provided an avenue for the students to apply the other three skills to their training."
Harrison et al. (2024)	No change	*Dependent variables targeted: awareness, non-judgement, refocusing, and action and acceptance*<br><br>Non-judgement<br>No statistically significant changes were seen for way dancers critically judge their own performance (P=.09)<br>Refocusing<br>No statistically significant changes were seen for how dancers refocus their attention from performance, (P=.75)<br>Awareness<br>No statistically significant changes were seen for the way dancers maintained awareness of their thoughts, feelings, and emotions, (P=.30)<br>Action and acceptance<br>No statistically significant changes were seen for how open dancers are toward experiences, (P=.57)	"Although dancers reported in the interviews that the sessions were valuable, they advised their ability to dedicate time to mindfulness ongoing would be challenging"
Jeong (2012)	No change	*Dependent variables targeted: flow (state and disposition), anxiety, imagery use, performance*<br><br>Flow:<br>No statistically significant changes were seen for flow (p > .05)<br>Flow state changed for both	No qualitative evidence available
Kaplan (2014)	Full change	*Dependent variables targeted: body image, eating attitudes, thin ideal, dance identity, self-esteem*<br><br>Body image:<br>Significant changes were seen for body image (p < .001)<br>Eating attitudes:<br>Significant changes were seen for eating attitude (p < .003)<br>Thin ideal:<br>Significant changes were seen for thin ideal (p < .001)<br>Dance identity<br>Significant changes were seen for dance identity (p < .001)<br>Self-esteem<br>Significant changes were seen for dance identity (p < .001).	No qualitative evidence available
Karin & Nordin-Bates (2	Moderate change	*Dependent variables targeted: creativity, perfectionistic cognitions, implicit and explicit sources of evaluation, intrinsic motivation*<br><br>Creativity<br>Significant increases for creativity (p < .03)<br><br>Perfectionistic cognitions<br>Significant increases for perfectionistic cognitions (p < .03)<br><br>Implicit sources of evaluation<br>Significant increases for implicit sources for evaluation (p < .01)<br>No statistically significant changes were seen for explicit sources of evaluation (p < .86)<br><br>Intrinsic motivation.<br>[illegible]	"Case study interviewees, representing the most and least perfectionistic students, reported heightened creativity, enjoyment and, in some cases, a strengthened sense of autonomy and self-regulation."
Klockare (2014)	Moderate change	*Dependent variables targeted: self-talk, emotional control, goal-setting, Imagery use, activation, relaxation, attentional control*<br><br>Self-talk<br>No statistically significant changes were seen for self-talk (p < .95)<br>Emotional control<br>No statistically significant changes were seen for self-talk (p < .81)<br>Goal-setting<br>No statistically significant changes were seen for self-talk (p < .69)<br>Imagery use<br>Significant changes were seen for self-talk (p < .31)<br>Activation<br>No statistically significant changes were seen for self-talk (p < .76)<br>Relaxation<br>Significant changes were seen for self-talk (p < .01)<br>Attentional control<br>Significant changes were seen for self-talk (p < .14)	No qualitative evidence available
Lubert et al. (2023)	Full change	*Dependent variables targeted: anxiety (trait performance anxiety, fear of negative evaluation,cognitive anxiety, somatic anxiety) self-efficacy, confidence, performance*<br><br>Anxiety<br>Significant changes were seen for trait anxiety (p < .010)<br>Significant changes were seen for fear of negative evaluation (p < .003)<br>Significant changes were seen for fear of cognitive anxiety (p < .001)<br>Significant changes were seen for fear of somatic anxiety (p < .006)<br><br>Self-efficacy<br>Significant changes were seen for self-efficacy (p < .008)<br>Confidence<br>Significant changes were seen for confidence (p < .002)<br>Performance<br>Significant changes were seen for performance quality (p < .013) but not for heart rate (p < 0.10)	"Participants were mostly content when looking back at the coaching process and their initial goals, but sometimes they also changed the goals themselves, which opened up new perspectives: "not to look for perfection or stability in every area, that 'now I'm prepared for everything', but also if I'm not prepared to something, that I can deal with it""
May et al. (2020)	No change	*Dependent variables targeted: creatvity, flexible thinking*<br><br>Creativity<br>No significant changes were seen for creativity (p = .098)<br><br>Flexible thinking<br>No significant changes were seen for flexible thinking (p = .079)<br>Significant changes were seen for flexible thinking in imagery group (p < .001.)	No qualitative evidence available
Moyle (2016)	No change	*Dependent variables targeted: mindfulness*<br><br>Mindfulness<br>No significant changes were seen for minfulness (no p value reported)	"To be honest I didn't really understand the point of mindfulness meditation at the beginning of the semester but now I feel of great benefit because of it. I now understand the different types of meditation and how powerful the brain can be– just rewording your intentions, how you think about things, or just focusing on a particular aspect helps you become calm and aware."
Noh et al. (2007)	Moderate change	*Dependent variables targeted: coping skills (coping with adversity, peaking under pressure, goal setting/mental preparation, concentration, freedom from worry, confidence and achievement motivation, coachability), injury*<br><br>Coping skills<br>Significant changes were seen for coping skills (p < .05.)<br><br>Injury<br>No significant changes were seen for injuries (p> .05)	No qualitative evidence available
Nordin-Bates et al. (202	No change	*Dependent variables targeted: self-esteem, self-compassion, perfectionism, disordered eating behaviors*<br><br>Self-esteem<br>No statistically significant changes were seen for self-esteem (p = .09)<br>Self-compassion<br>No statistically significant changes were seen for self-compassion (p = .80)<br>Perfectionism<br>No statistically significant changes were seen for perfectionism (p =.28; p =.86)<br>Disordered eating<br>No statistically significant changes were seen for disordered eating (p = .48)	"…one pupil mentioned how "I feel that I have learnt but that it is still hard to use"; another explained how s/he had learnt a lot, but "it isn't so easy to change behaviours… so quickly". They also felt that the time taken for workshops sometimes conflicted with their need for free time, or that the time could have been better spent."
Petsilas & Massarelli (2	Moderate change	No quantitative evidence available	Targeted outcome: improving body perception (increased body relaxation, awareness of tension and injuries, enhanced sense of groundedness), self-awareness and concentration (increased clarity of thoughts and emotions, decreased self-judgement, enhanced self-regulation, coping skills for perceived stress and anxiety, increased self-acceptance and confidence<br><br>"All the participants noticed positive effects on their body perception (increased body relaxation, awareness of tension and injuries, enhanced sense of groundedness), self-awareness and concentration (increased clarity of thoughts and emotions, decreased self-judgement, enhanced self-regulation, coping skills for perceived stress and anxiety, increased self-acceptance and confidence)."
Ritchie & Brooker (2018	Moderate change	No quantitative evidence available	Targeted outcome: imrpoving imagery use<br><br>"The articulation of the spine I'm pretty sure it got better … So compared to the first sessions when I wasn't really understanding the journey that the spine must make and I wasn't really understanding how to use the articulation of the spine … But when you finally focus on that and understand the way it helps you … Visualising. Really I mean really I know it's a very obvious reason but actually visualising the spine curving with every movement is actually amazing!"
Santos et al. (2022)	Moderate change	*Dependent variables targeted: well-being, coping, depression, self-concept*<br><br>Well-being<br>No statistically significant changes were seen for well-beingg (p <.804)<br>Coping<br>No statistically significant changes were seen for coping (p <.455)<br>Depression<br>Significant changes were seen for depression (p < .035)<br>Self-concept<br>No statistically significant changes were seen for self-concept (p <.406)	No qualitative evidence available
Skvarla & Clement (201	No change	*Dependent variables targeted: Coping (coping with adversity, peaking under pressure, goal setting/mental preparation, concentration, freedom from worry, confidence and achievement motivation, coachability), injury*<br><br>Coping<br>No statistically significant changes were seen for coping (p <.326)<br><br>Injury<br>No statistically significant changes were seen for injuries:<br>the intensity of pain was reported as minor (N = 41), moderate (N = 42), and severe (N = 3) levels.)	"I do not do well with mindfulness or just relaxing because I will just either fall asleep or my mind will go everywhere."
Stackpole & Quiroga-Ga	Full change	Dependent variables targeted: performance anxiety, anxiety credibility, self-perceived anxiety<br><br>Performance anxiety<br>Significant changes were seen for depression (p <0.001)<br>Anxiety credibility<br>Significant changes were seen for depression (p <0.001)<br>Self perceived anxiety<br>Significant changes were seen for depression (p <0.001)	The qualitative results demonstrated that the participants could identify problems—distancing themselves from them, transforming negative thoughts into positive ones, and getting to know/broadening their resources to face anxious situations.
Zhang (2022)	Change achieved: Not specified	Not specified	Not specified

**Summary**	**Change achieved**	**Quantitative evidence**	**Qualitative evidence**
	Not specified N= 2 (2/21) Andersen, 2009; Zhang, 2022<br><br>No change achieved N= 7 (7/21) (Fryer, 2018; Harrison et al., 2024; Jeong, 2012; May et al., 2020; Moyle, 2016; Nordin-Bates et al., 2023; Skvarla & Clement, 2019).<br><br>Moderate change achieved N= 9 (9/21) (Carattini, 2020; Castillo, 2018; Gerena, 2015; Karin & Nordin-Bates, 2020; Klockare, 2014; Noh et al., 2007; Petsilas & [illegible]	Quantitative results from quantiative designs:<br>N= 7 (Castillo, 2018; Jeong, 2012; Kaplan, 2014; Klockare, 2014; May et al., 2020; Noh et al., 2007; Santos et al., 2022))<br><br>Quantitative results from mixed methods designs N= 9 (Fryer, 2018; Gerena, 2015; Harrison et al., 2024; Karin & Nordin-Bates, 2020; Lubert et al., 2023; Moyle, 2016; Nordin-Bates et al., 2023; Skvarla & Clement, 2019; Stackpole & Quiroga-Garza, 2023)<br><br>**In-between studies results**<br><br>*Of the fully effective studies with quantitative evidence N= 3 (3/21) (Kaplan, 2014; Lubert et al., 2023; Stackpole & Quiroga-Garza, 2023)*<br><br>2 (2/3) described changes in mental health outcomes (e.g., anxiety, depression, well-being) (Lubert et al., 2023; Stackpole & Quiroga-Garza, 2023),<br><br>1 (1/3) described changes in mental qualities (e.g., self-awareness, self-esteem, creativity) (Kaplan, 2014).<br><br>*Of the moderately effective studies with quantitative evidence N= (6/9) (Castillo, 2018; Gerena, 2015; Karin & Nordin-Bates, 2020; Klockare, 2014; Noh et al., 2007; Santos et al., 2022)*<br><br>3 (3/6) described changes in mental qualities (Castillo, 2018; Karin & Nordin-Bates, 2020; Noh et al., 2007)<br><br>1 (1/6) reported changes in mental health outcomes (Santos et al., 2022)<br><br>[illegible]	**Qualitative evidence**<br>Qualitative evidence from qualitative designs N= 5 (Andersen, 2009; Carattini, 2020; Petsilas & Massarelli, 2024; Ritchie & Brooker, 2018; Zhang, 2022)<br><br>Qualitative evidence form mixed methods designs N= 9 (Fryer, 2018; Gerena, 2015; Harrison et al., 2024; Karin & Nordin-Bates, 2020; Lubert et al., 2023; Moyle, 2016; Nordin-Bates et al., 2023; Skvarla & Clement, 2019; Stackpole & Quiroga-Garza, 2023)<br><br>**In-between studies results**<br><br>*Of the fully effective studies with qualitative evidence N= 2* (2/3) (Lubert et al., 2023; Stackpole & Quiroga-Garza, 2023)<br><br>2 (2/3) described changes in mental health outcomes (e.g., anxiety, depression, well-being) (Lubert et al., 2023; Stackpole & Quiroga-Garza, 2023)<br><br>*Of the moderately effective studies with qualitative evidence N=* 5 (5/9) (Carattini, 2020; Gerena, 2015; Karin & Nordin-Bates, 2020; Petsilas & Massarelli, 2024; Ritchie & Brooker, 2018)<br><br>3 (3/5) described changes in mental qualities (Karin & Nordin-Bates, 2020; Petsilas & Massarelli, 2024; Ritchie & Brooker, 2018)<br><br>1 (1/5) reported changes in mental health outcomes (Carattini, 2020)<br><br>1 (1/5) changes in dependent variables measuring use of mental techniques (e.g., imagery, self-talk) (Gerena, 2015).
