# Supplementary material for: Mapping characteristics of mental skills training interventions in dance using TIDieR: a mixed-methods systematic review
Source: BMJ Open. 2025 Nov 13;15(11):e104552. doi: 10.1136/bmjopen-2025-104552 (PMC12625948; doi:10.1136/bmjopen-2025-104552)
Supplement: online supplemental table 2 [file bmjopen-15-11-s002.pdf]

Table S2

Reference	Title	Are there clear research questions?	Do the collected data allow to address the research questions?	Qualitative 1: Is the qualitative approach appropriate to answer the research question?	Qualitative 2: Are the qualitative data collection methods adequate to address the research question?	Qualitative 3: Are the findings adequately derived from the data?	Qualitative 4: Is the interpretation of results sufficiently substantiated by data?	Qualitative 5: Is there coherence between qualitative data sources, collection	Quantitative RCT 1: Is randomization appropriately performed?	Quantitative RCT 2: Are the groups comparable at baseline?	Quantitative RCT 3: Are there complete outcome data?	Quantitative RCT 4: Are outcome assessors blinded to the intervention provided?	Quantitative RCT 5: Did the participants adhere to the assigned intervention?	Quantitative non randomized 1: Are the participants representative of the target population?	Quantitative non randomized 2: Are measurements appropriate regarding both the outcome and intervention (or	Quantitative non randomized 3: Are there complete outcome data?	Quantitative non randomized 4: Are the confounders accounted for in the design and analysis?	Quantitative non randomized 5: During the study period, is the intervention administered (or exposure occurred) as intended?	Mixed methods 1: Is there an adequate rationale for using a mixed methods design to address the research question?	Mixed methods 2: Are the different components of the study effectively integrated to answer the research question?	Mixed methods 3: Are there outputs of the integration of qualitative and quantitative components adequately interpreted?	Mixed methods 4: Are divergences and inconsistencies between quantitative and qualitative results adequately addressed?	Mixed methods 5: Do the different components of the study adhere to the quality criteria of each tradition of the methods involved?	Quality assessment (LoE and CerQual)
Andersen 2009	The "canon" of psychological skills training for enhancing performance	No	Can't tell	Can't tell	Can't tell	Can't tell	Yes	No																Low confidence
Carattini 2020	Psychological skills in ballet training: An approach to pedagogy for the fulfilment of student potential	Yes	Yes	Can't tell	Yes	Yes	Yes	Yes																High confidence
Castillo 2019	Implementation and evaluation of a performance profile intervention with collegiate dancers: A randomized controlled trial	Yes	Yes						Can't tell	Yes	Yes	No	Can't tell											Moderately high risk of bias (3)
Fryer 2019	The cascading effect: Mitigating the effects of choking under pressure in dancers	Yes	Yes																No	No	No	No	Yes	Moderately high risk of bias/ High confidence
Gerena 2015	Positive thinking in dance: The benefits of positive self-talk practice in conjunction with somatic exercises for collegiate dancers	Yes	Yes																No	Can't tell	Yes	No	No	High risk of bias / Moderate confidence
Harrison 2024	A 6-week mindfulness-acceptance-commitment intervention to improve professional ballet dancers' psychological states around performance	Yes	Yes																No	Can't tell	No	No	No	Moderately high risk of bias / Moderate confidence
Jeong 2012	The application of imagery to enhance'flow state'in dancers	Yes	Yes						Can't tell	Yes	Can't tell	No	Can't tell											High risk of bias (4)
Kaplan 2014	Gaining control of the dancer in the mirror: A prevention program for recreational ballet students	Yes	Yes						Can't tell	Yes	Yes	No	Can't tell											High risk of bias (4)
Karin 2020	Enhancing creativity and managing perfectionism in dancers through implicit learning and sensori-kinetic imagery	Yes	Yes																No	Yes	Yes	Yes	No	Moderately high risk of bias / Moderate confidence
Klockare 2014	A psychological skills training program for dancers: Evaluation of the dancers' use of psychological skills training techniques and possible effects of the program	Yes	Yes											Yes	Yes	No	No	Can't tell						High risk of bias (4)
Lubert 2023	Effects of tailored interventions for anxiety management in choking-susceptible performing artists: a mixed-methods collective case study	Yes	Yes																Yes	Yes	Yes	Yes	Can't tell	Moderately high risk of bias/ High confidence
May 2020	Enhancing creativity by training metacognitive skills in mental imagery	Yes	Yes											No	Yes	No	Yes	Can't tell						High risk of bias (4)
Moyle 2016	Mindfulness and dancers	Yes	Can't tell																Can't tell	Can't tell	Can't tell	Yes	Can't tell	High risk of bias / Moderate confidence
Noh 2007	Psychological intervention programs for reduction of injury in ballet dancers	Yes	Yes											Can't tell	No	Can't tell	Yes	Can't tell						High risk of bias (4)
Nordin-Bates 2023	Preventing disordered eating in teenage ballet students: Evaluation of dancexcellent, a combined CBT and nutrition education intervention	Yes	Yes																No	No	Yes	Can't tell	Yes	Moderately high risk of bias / Moderate confidence
Petsilas 2024	Evaluating the impact and effectiveness of meditation in a dance conservatoire context	Yes	Yes	Yes	Yes	Can't tell	Yes	Yes																Moderate confidence
Ritchie 2018	Imaging the future: an autoethnographic journey of using a guided and cognitive-specific imagery intervention in undergraduate release-based contemporary dance technique	Yes	Can't tell	Yes	Yes	Can't tell	No	Can't tell																Low confidence
Santos 2022	"Performing emotions and suffering": Recovery of depressive symptoms involving adolescent intervention in art and dance schools	Yes	Yes											Yes	Yes	Can't tell	Yes	Can't tell						Moderately high risk of bias (3)
Skvarla 2019	The delivery of a short-term psychological skills Training program to college dance students: A pilot study examining coping skills and injuries	Yes	Yes																No	Can't tell	Yes	No	Yes	Moderately high risk of bias/ Moderate confidence
Stackpole 2023	Overcoming stage anxiety with a solution-focused approach	No	No																No	No	Can't tell	No	Can't tell	High risk of bias/ Low confidence
Zhang 2022	Psychological interventions to performance enhancement in dance: A hypothetical case study	Yes	Can't tell	Can't tell	Yes	Can't tell	No	Can't tell																Low confidence

Summary

Clear research question
N= 19
Yes (n= 19) (Carattini, 2020; Castillo, 2018; Fryer, 2018; Gerena, 2015; Harrison et al., 2024; Jeong, 2012; Kaplan, 2014; Karin & Nordin-Bates, 2020; Klockare, 2014; May et al., 2020; Noh et al., 2007; Lubert et al., 2023; Moyle, 2016; Nordin-Bates et al., 2023; Petsilas & Massarelli, 2024; Ritchie & Brooker, 2018; Santos et al., 2022; Zhang, 2022)

No (n=2) (Andersen, 2009; Stackpole & Quiroga-Garza, 2023)

Reasons: Unclear aim or hypothesis stated (n=2)

Data that answers question
Yes (n = 16) (Carattini, 2020; Castillo, 2018; Fryer, 2018; Gerena, 2015; Harrison et al., 2024; Jeong, 2012; Kaplan, 2014; Karin & Nordin-Bates, 2020; Klockare, 2014; May et al., 2020; Noh et al., 2007; Lubert et al., 2023; Nordin-Bates et al., 2023; Petsilas & Massarelli, 2024; Santos et al., 2022)

No (n=5) (Andersen, 2009; Moyle, 2016; Ritchie & Brooker, 2018 Stackpole & Quiroga-Garza, 2023; Zhang, 2022)

Reason 1: insufficient information on whether data was sufficient to answer the research question

Qualitative summary:
N = 5

Between study analysis

3 studies (3/5) could not substantiate if a qualitative approach was appropriate to answer the research question (Andersen, 2009; Carattini, 2020; Zhang, 2022) or whether there is coherence between data sources, collection analysis and interpretation (3/5) (Andersen, 2009; Ritchie & Brooker, 2018; Zhang, 2022).

Consequently, 3 studies (3/5) elicited low confidence in their findings (Andersen, 2009; Ritchie & Brooker, 2018; Zhang, 2022), 1 study (1/5) moderate confidence (Petsilas & Massarelli, 2024) and 1 investigation (1/5) high confidence in the presented findings (Carattini, 2020).

Quantitative random summary
N= 3

Between study analysis

None of the 3 randomized studies (3/3) neither appropriately performed nor adequately reported whether the participants adhered to the intervention (Castillo, 2018; Jeong, 2012; Kaplan, 2014).

This resulted in 2 studies (2/3) having a high risk of bias (Jeong, 2012; Kaplan, 2014)

1 (1/3) having a moderately high risk of bias (Castillo, 2018).

Quantitative non random summary
N= 4

Between study analysis

None of the 4 non-randomized studies (4/4) (Klockare, 2014; May et al., 2020; Noh et al., 2007; Santos et al., 2022) presented complete outcome data nor could it determined whether the intervention was administered as intended.

Mixed methods summary
N= 9

Between study analysis

Overall 8 of mixed methods studies (8/9) did not enclose an adequate rationale for using a mixed methods design (Fryer, 2018; Gerena, 2015; Harrison et al., 2024; Karin & Nordin-Bates, 2020; Moyle, 2016; Nordin-Bates et al., 2023; Skvarla & Clement, 2019; Stackpole & Quiroga-Garza, 2023).

7 (7/9) either did not integrate the qualitative and quantitative components adequately (3/7) (Fryer, 2018; Nordin-Bates et al., 2023; Stackpole & Quiroga-Garza, 2023) or it could not be determined, whether researchers integrated components effectively to answer the research question (4/7) (Gerena, 2015; Harrison et al., 2024; Moyle, 2016; Skvarla & Clement, 2019).

Qualitative
3 of the qualitative studies (3/5) elicited low confidence in their findings (Andersen, 2009; Ritchie & Brooker, 2018; Zhang, 2022), 1 study (1/5) moderate confidence (Petsilas & Massarelli, 2024) and 1 investigation (1/5) high confidence in the presented findings (Carattini, 2020).

Quantitative non-randomized
The strength of evidence indicated that 3 of the non-ranomized studies (3/4) had a high risk of bias (Klockare, 2014; May et al., 2020; Noh et al., 2007) and 1 (1/4) had a moderately high risk of bias (Santos et al., 2022).

Quantitative randomized
Of the 3 randomized studies, 2 (2/3) had a high risk of bias (Jeong, 2012; Kaplan, 2014) and 1 (1/3) had a moderately high risk of bias (Castillo, 2018).

Mixed methods
General challenges to report divergences and inconsistencies between qualitative and quantitative results (6/9) (Fryer, 2018; Gerena, 2015; Harrison et al., 2024; Nordin-Bates et al., 2023; Skvarla & Clement, 2019; Stackpole & Quiroga-Garza, 2023) and a lack of consideration for quality criteria of the methods involved (6/9) (Gerena, 2015; Harrison et al., 2024; Karin & Nordin-Bates, 2020; Lubert et al., 2023; Moyle, 2016; Stackpole & Quiroga-Garza, 2023) resulted in general moderately high to high risk of bias for the mixed methods studies in this review.
