# Supplementary material for: Mapping characteristics of mental skills training interventions in dance using TIDieR: a mixed-methods systematic review
Source: BMJ Open. 2025 Nov 13;15(11):e104552. doi: 10.1136/bmjopen-2025-104552 (PMC12625948; doi:10.1136/bmjopen-2025-104552)
Supplement: Supplementary file 6 [file bmjopen-2025-104552.reviewer_comments.pdf]

PEER REVIEW HISTORY

BMJ Open publishes all reviews undertaken for accepted manuscripts. Reviewers are asked to complete a checklist review form and are provided with free text boxes to elaborate on their assessment. These free text comments are reproduced below.

ARTICLE DETAILS

Title (Provisional)

Mapping characteristics of mental skills training interventions in dance using TIDieR: A mixed-methods systematic review

Authors

Dwarika, Michelle Schachtler; Quinton, Mary L; Nordin-Bates, Sanna; Cumming, Jennifer

---

VERSION 1 - REVIEW

**Reviewer**	**1**
**Name**	**Benoît-Piau, Justine**
**Affiliation**	**Amsterdam UMC Locatie VUmc**
**Date**	**28-May-2025**
**COI**	**None**

General

This is a a mixed methods systematic review that aims to provide an overview of existing mental skills training in dance and examine their characteristics and effectiveness. I have reviewed the protocol for this manuscript in 2024.

After reviewing the manuscript, I think that it needs a major re-write before it could be published. The main reasons are 1) the results and discussion seem to overlap, 2) results section would greatly benefit from being re-centered around the objectives 3) the quantitative to qualitative data translation is not clearly described (i.e. when it was used and how it was done), 4) the objectives are not answered properly in the results section, and 5) the entire structure of the manuscript makes it difficult to understand the key messages.

Please review the entire manuscript for punctuation. There are question marks in the middle of sentences, dots and commas one after the other, missing dots at the end of sentences, unnecessary spaces, etc.

Abstract

- Please add a dot at the end of your objectives sentence.

- Results do not seem to reflect objectives.

Introduction

- First paragraph is very clear, the definition of mental qualities/skills/techniques was very enlightening.

- I believe the 2nd, 3rd and 4th paragraph could be shortened to improve clarity and be more to the point.

Methods

- The outcome section of PICO appears vague to me. Were there other changes in performance?

- Data extraction section, last two sentences : « After consultation » of whom? Was this because the request for missing data was not answered?

Results

- This section is extremely long and, in my opinion, contains unnecessary detailed information. I believe the TIDieR checklist section would greatly benefit from being shortened. At the moment, I cannot identify what is important because it is simply too extensive.

- The effectiveness section seems under-described. How was effectiveness measured? What would qualify as moderately effective? Which outcomes were studied?

- After reading the results section, I don't have an answer to objectives 1 and 3.

Discussion

- RQ1 : I feel like what is discussed here is not really presented in the results. In fact, I think lines 3-12 belong in the results more than the discussion.

- RQ2 : Again, I feel like lines 1-6 belong in the results more than the discussion.

- RQ3 : Similarly, the first paragraph seems more like results than discussion.

- Limitations should include the fact that the studies all had moderate or high risk of bias… which influences the strength of your findings.

Hope these comments help.

**Reviewer**	**2**
**Name**	**Ambegaonkar, Jatin P.**
**Affiliation**	**George Mason Univ**
**Date**	**14-Jul-2025**

COI None

Forward head posture prevalence in collegiate dance majors as measured by a posture analysis app

OVERALL

This article examines a relatively underserved population - dancers

However- there are multiple areas where clarification is needed. Also in several sections, suggest subheadings and smaller paragraphs to help readability.

SPECIFIC

TITLE: Can be more directional - consider telling the reader what you found to make it more interesting.

ABSTRACT

What does researchers showed great intuition mean in this context? Are authors congratulating themselves in the abstract?

Conclusion - Provide more specific information e.g.. what does the manuscript add to the literature

Strengths

1 = Add ‘To our knowledge’ - you do state this in the discussion well

2 = This is a general statement - add specifics for readers

3 = Again - see point 2, as a reader, I should be able to get some good information here not generally told that the TIDieR checklist is good

Limitation

1 = No that was a delimitation - yes - do not worry - state it as is and advence

2 = Only USA ? Or overall western ?

INTRODUCTION

Page 8 line 12 - what does manualized mean in this context?

METHODS

Well done

RESULTS

Clear. May consider adding some tables in the manuscript and not in supplementary material as per journal limitations

DISCUSSION

Well done. Consider a section to guide further research with specific recommendations

CONCLUSIONS

What are the key findings. These should be consistent and relate to the study strengths and the abstract

Tables and Figures:

Tables - Well done

Figures - Well done

- consider a more contrasting and consistent color scheme for Fig 2 and 3

VERSION 1 - AUTHOR RESPONSE

Reviewer 1	Please review the entire manuscript for punctuation. There are question marks in the middle of sentences, dots and commas one after the other, missing dots at the end of sentences, unnecessary spaces, etc	The whole manuscript has been checked and reviewed for spelling mistakes, missing dots and unnecessary spaces.
Reviewer 1	Abstract<br>Please add a dot at the end of your objectives sentence.	A dot has been added at the end of the objectives sentence in line 30.
Reviewer 1	Results do not seem to reflect objectives.	Thank you for making us aware of this inconsistency. We can see that the phrasing of examining "characteristics" was vague and not properly covered in the review. Therefore, we have adjusted the aims in the objective to reflect the results more accurately in lines 27-30. This now reads as follows:<br><br>"Therefore, the aim of this mixed-methods systematic review (MMSR) was to provide an overview of existing MST interventions in dance and examine (1) the characteristics of effective mental skills training interventions in dance and (2) how the reporting of these interventions aligned with the 12 TIDiER items."
Reviewer 1	Introduction<br>First paragraph is very clear, the definition of mental qualities/skills/techniques was very enlightening.<br>I believe the 2nd, 3rd and 4th paragraph could be shortened to improve clarity and be more to the point	Thank you for letting us know that the definition was clear, which was great to hear.<br><br>We agree with your other comment and have cut the introduction considerably (see lines 104-132).

Reviewer 1	Methods<br>The outcome section of PICO appears vague to me. Were there other changes in performance?	We have amended this point in the manuscript (see lines 183-184), which now reads:<br><br>"*Relevant studies examined pre- to post-intervention changes in mental qualities, mental health, and/or symptoms of common mental disorders (e.g., depression, anxiety).*"
Reviewer 1	Data extraction section, last two sentences : « After consultation » of whom? Was this because the request for missing data was not answered?	We have now clarified this point in the re-worked manuscript. The author had not published the intervention data of that particular study (due to it being part of a thesis). After conversations between us and that author, we concluded together that it would be best to exclude the study from the review. We have tried to reflect this decision by writing in the manuscript (see lines 232-233):<br><br>*"One author was contacted to request missing data. However, due to data not having been formally published, the study was excluded from the review."*
Reviewer 1	Results<br>This section is extremely long and, in my opinion, contains unnecessary detailed information. I believe the TIDieR checklist section would greatly benefit from being shortened. At the moment, I cannot identify what is important because it is simply too extensive.	We have made the TIDieR section more concise and cut a considerable amount of information from this section. However, we still want to retain this section as a point of reference for readers as well as to maximize the number of findings that could be reported . Therefore, we think that the re-worked manuscript honors your feedback while staying true to our endeavor to offer valuable insights to researchers and practitioners.
Reviewer 1	The effectiveness section seems under-described. How was effectiveness measured? What would	Thank you for this thoughtful comment. We have answered each question in turn:

	qualify as moderately effective? Which outcomes were studied?	1. How was effectiveness measured?<br><br>To make this information clearer, we have amended the manuscript to the following (see lines 309-314):<br><br>*Two of the included studies (2/21) did not specify whether the intervention was effective. Three studies (3/19) reported full change in one or more dependent variable from pre- to post intervention (significant p value of <0.5 in quantitative studies or equivalent description in qualitative studies). 9 studies (9/19) reported moderate effectiveness (i.e., one or more dependent variables significantly changing from pre-to post-intervention in quantitative studies as reflected in a p value of <0.5 or in or equivalent description in qualitative studies). 7 (7/19) studies reported no change in the dependent variables.*<br><br>2. What would qualify as moderately effective<br><br>We see how this point could be confusing, considering that not all readers will have read the protocol where we defined effectiveness more precisely. We have now added the information in the manuscript (see response to the first question above).<br><br>3. Which outcomes were studied?<br><br>These outcomes are described in the manuscript as follows (see lines 320-324):<br><br>*"Of the studies achieving full changes, 2 (2/3) described changes in mental health outcomes (e.g., anxiety,*

		depression, well-being), and 1 (1/3) described changes in mental qualities (e.g., self-awareness, self-esteem). Of the moderately effective studies, 5 (5/9) described changes in mental qualities, 2 (2/9) reported changes in mental health outcomes, and 2 (2/9) measured changes in use of mental techniques (e.g., imagery, self-talk) as the dependent variables".
Reviewer 1	After reading the results section, I don't have an answer to objectives 1 and 3.	We agree with your comment and think it was a point well made. To reflect your feedback, we have adapted the aims to the result section and cut the third aim to make the manuscript shorter and to the point. The aims are as re-worded as follows (lines 134-139):<br><br>*"The aim of this MMSR was to provide an overview of existing MST interventions in dance and examine their characteristics and effectiveness within the TIDieR framework. To achieve this objective, the following was investigated:*<br><br>*1. What are the characteristics of effective mental skills training interventions in dance?*<br>*2. How does the reporting of these interventions align with the 12 TIDiER items and what, if any, reporting gaps are occurring?"*

		Consequently, we have also made changes to the results section. We agree with you that the effectiveness section benefits from being elaborated on. Therefore, we have moved certain parts from the discussion section to the results section and tried to foreshadow some of the discussion points to the reader in an additional, short effectiveness paragraph (see lines 326-339).
Reviewer 1	Discussion<br>RQ1 : I feel like what is discussed here is not really presented in the results. In fact, I think lines 3-12 belong in the results more than the discussion.	Thank you for making us aware of this. We agree with you that the discussion and result section overlapped. We have cut down on the discussion (see lines 439-499) and results sections (see lines 308-437) and re-focused on the two aims we have identified.
Reviewer 1	RQ2 : Again, I feel like lines 1-6 belong in the results more than the discussion.	Agreed. We have addressed this in the new manuscript and re-worked the discussion section according to this feedback (see lines 326-339).
Reviewer 1	RQ3 : Similarly, the first paragraph seems more like results than discussion	Agreed and re-worded this text as explained in the above comment. As visible in lines 447 to 471, we have moved or cut text to diminish the overlap between results and discussion.
Reviewer 1	Limitations should include the fact that the studies all had moderate or high risk of bias… which influences the strength of your findings	As suggested, this has been added to the limitations section (see lines 529-532).
Reviewer 2	in several sections, suggest subheadings and smaller paragraphs to help readability.	This is a great point. As per the suggestion of Reviewer 1, we have cut some text and hope that this will help with readability. As the text has been made more concise, we think that the existing subheadings will be sufficient

		to guide the reader through the manuscript.
Reviewer 2	TITLE<br>Can be more directional - consider telling the reader what you found to make it more interesting	Thank you for your suggestion. We agree and have changed the title to:<br><br>*"Mapping characteristics of mental skills training interventions in dance using TIDieR: A mixed methods systematic review"*
Reviewer 2	ABSTRACT<br>What does researchers showed great intuition mean in this context? Are authors congratulating themselves in the abstract?	Thank you for pointing this out, and it was certainly not meant as such. We now see that the phrasing may have been confusing to the reader! Therefore, we have cut this part in the abstract and tried to be clearer and more concise in describing the results (see lines 45-49). It now reads:<br><br>*"Findings from the 21 included studies indicated that intervention effectiveness was determined by a range of characteristics relating to both recipients (e.g., dancers' needs, values and outcomes) and deliverers (e.g., delivery styles and perspectives, interaction between situation and context) of interventions. While TIDieR items describing intervention content were the most reported, only a few studies described fidelity."*
Reviewer 2	Conclusion - Provide more specific information e.g.. what does the manuscript add to the literature	This has been addressed in the new manuscript and added to the conclusion section in the abstract (see lines 54-58).

Reviewer 2	Strengths<br>1 = Add 'To our knowledge' - you do state this in the discussion well	This has been addressed as suggested (see line 65).
Reviewer 2	2 = This is a general statement - add specifics for readers	We agree and have changed all mentioned strengths (as suggested by you and the editor), see lines 64-71.
Reviewer 2	3 = Again - see point 2, as a reader, I should be able to get some good information here not generally told that the TIDieR checklist is good	*"Thus, by offering an overview of existing MST interventions and using TIDieR to disseminate and understand existing characteristics, this MMSR not only addresses gaps in dance literature but also offers researchers and practitioners lessons learned to enhance the planning, conducting and reporting of their interventions."*
Reviewer 2	Limitation<br>1 = No that was a limitation - do not worry - state it as is an advantage	Thank you for making us aware of this. As suggested by the editor, we have changed the strengths and limitations section and focused all points on methodological strengths and shortcomings (see lines 73-76).
Reviewer 2	2 = Only USA ? Or overall western ?	This has been adjusted and USA removed (see lines 74-76).
Reviewer 2	INTRODUCTION<br>Page 8 line 12 - what does manualized mean in this context?	As suggested by Reviewer 1, we have cut some text in the introduction to make this section more concise. We have addressed your question and reformulated that part of the text (see lines 104-119).
Reviewer 2	METHODS<br><br>Well done	Thank you and we appreciate your positive comment about our work! Due to the points made by Reviewer 1, we have adapted parts of the text in the method section (see lines 183-184; lines 232-233; lines 253-255) to

		address the feedback given, which we believe has improved the methods further.
Reviewer 2	RESULTS<br>Clear. May consider adding some tables in the manuscript and not in supplementary material as per journal limitations	We have added two tables in the manuscript to aid the reader in seeing how effectiveness was determined (as shown in table 2, added in line 341) and how the different evidence was brought together (as shown in table 3, added in line 436).
Reviewer 2	DISCUSSION<br>Well done. Consider a section to guide further research with specific recommendations	A new section on recommendations and future research has been added (see lines 500-515).
Reviewer 2	CONCLUSIONS<br>What are the key findings. These should be consistent and relate to the study strengths and the abstract	The conclusion section has been re-worded and more explicitly relates back to the strengths and the abstract (see lines 534-545).
Reviewer 2	Tables - Well done	Thank you. We are happy that you liked the tables and that they clearly conveyed the information to the reader.
Reviewer 2	Figures - Well done<br>consider a more contrasting and consistent color scheme for Fig 2 and 3	As suggested, we have adjusted the color scheme for Figure 2 (see line 279) and 3 (see line 297).

VERSION 2 - REVIEW

Reviewer **1**

Name **Benoît-Piau, Justine**

Affiliation **Amsterdam UMC Locatie VUmc**

Date **13-Oct-2025**

COI

General

This is a a mixed methods systematic review that aims to provide an overview of existing MST interventions in dance and examine their characteristics and effectiveness.

Abstract

- The TIDieR objective seems to come out of nowhere. Would it be possible to shorten the conclusion and add a sentence on why TIDieR is relevant here in the objectives section?

- Also, if you have some space, please describe shortly the TIDieR framework as it is central to the study.

Introduction

- Thank you for shortening the introduction. The importance of the TIDieR framework is also more clearly explained.

Methods

- Thank you for the adjustments.

Results

- Line 310: What do you mean by "full" change?

- I still believe the TIDieR section is too long. It feels like most information could be relayed in a table, with specific details described in the text, resulting in a much more concise section.

- Line 433: repoted > reported.

Discussion

- Thank you for restructuring.

- Lines 476-479: Everything that is a description of the items should be in the Methods.

- RQ2 seems very focused on fidelity. Are there other aspects that should be discussed given that the results don't focus solely on fidelity?

VERSION 2 - AUTHOR RESPONSE

Who	Comment	Reply to reviewers
Reviewer 1	Abstract<br>The TIDieR objective seems to come out of nowhere. Would it be possible to shorten the conclusion and add a sentence on why TIDieR is relevant here in the objectives section?	We agree and have adjusted the abstract to incorporate the suggestion made, see line 26-29.
Reviewer 1	Also, if you have some space, please describe shortly the TIDieR framework as it is central to the study.	We have explained the TIDieR framework as concisely as possible in the modified sentence in line 26-29. However, we are aware that abstracts should not succeed the 300 word limit and needed to make careful considerations what to cut while not eliminating other points added by reviewer 2 in the previous reviewing round
Reviewer 1	Introduction<br>Thank you for shortening the introduction. The importance of the TIDieR framework is also more clearly explained.	We are glad that the introduction and the importance of the TIDieR framework have become clearer.
Reviewer 1	Methods<br>Thank you for the adjustments.	It is great to hear that you liked the adjustments made to the methods section
Reviewer 1	Results<br>- Line 310: What do you mean by “full” change?	We see how this could be unclear to the reader and have adjusted the wording to “achieving intended outcomes” in lines 304, 309 and 315, 321
Reviewer 1	- I still believe the TIDieR section is too long. It feels like most information could be relayed in a table, with specific details described in the text, resulting in a much more concise section	We have followed your suggestion to cut back the TIDieR items from the results section and instead we have created a table (Table 3) with a concise overview of these results. We have added a short text description explaining the results in the table in lines 348-359.
Reviewer 1	- Line 433: repoted > reported.	Thank you for spotting this. We have amended the mistake in line 361.

Who	Comment	Reply to reviewers
Reviewer 1	Discussion<br>- Thank you for restructuring.	We are glad that you like how the discussion has been restructured.
Reviewer 1	Lines 476-479: Everything that is a description of the items should be in the Methods.	Agreed. However, since this is a description of the reporting from the included studies rather than a description of the TIDieR items themselves, we have moved this description to lines 340-343 in the result section.
Reviewer 1	- RQ2 seems very focused on fidelity. Are there other aspects that should be discussed given that the results don't focus solely on fidelity?	We have tried to balance the discussion of fidelity with the use of MST in the intervention content and have therefore adjusted the discussion of RQ2 in lines 399-414.
